# Advances in Genetic Engineering Techniques for Improved Forest Trees: Applications in Biomass, Stress Resilience and Carbon Sequestration

**DOI:** 10.3390/ijms262010192

**Published:** 2025-10-20

**Authors:** Sophia Hydarry Matola, Jingjing Li, Meiou Sun, Lu Yang, Wenhui Zhuang, Jingli Yang

**Affiliations:** 1 State Key Laboratory of Tree Genetics and Breeding, Northeast Forestry University, Harbin 150040, China; 2023420031@nefu.edu.cn (S.H.M.); 2022020033@nefu.edu.cn (J.L.); 2023120065@nefu.edu.cn (L.Y.); monqi@nefu.edu.cn (W.Z.); 2 Liangshui Experimental Forest Farm, Northeast Forestry University, Yichun 153000, China; sh2013@nefu.edu.cn

**Keywords:** molecular design breeding, CRISPR-Cas9, lignin engineering, drought tolerance mechanisms, phytoremediation, carbon sequestration, gene flow risks, sustainable forestry

## Abstract

Forest biotechnology is rapidly advancing from conventional breeding toward molecular design, enabling the development of genetically modified trees (GMTs) with traits such as accelerated growth, stress resilience, and improved wood properties. This review systematically examines recent breakthroughs in tree genetic engineering, beginning with traditional methods and progressing to CRISPR-based precision editing and multi-omics-guided trait design. We highlight applications in wood quality (e.g., lignin reduction in *Populus* spp.), drought tolerance (e.g., PagHyPRP1 and *PtoMYB142* editing), phytoremediation (e.g., heavy metal accumulation in poplar), and carbon sequestration. We also evaluate ecological and socio-regulatory challenges, including gene flow risks and public acceptance. Based on this integrated analysis, we outline future directions for responsible deployment of GMTs in sustainable forestry and global carbon neutrality efforts.

## 1. Introduction

**Figure 1:**
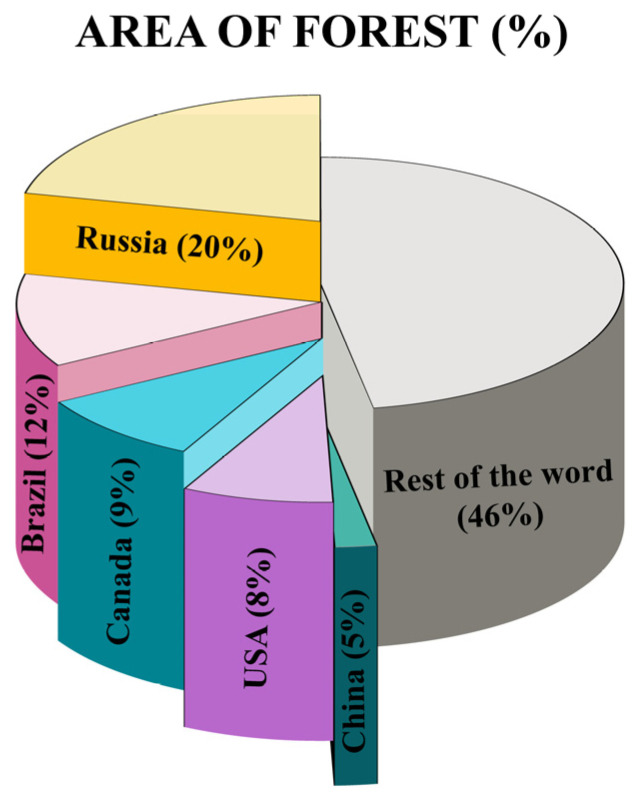
Forest cover percentage worldwide.

Forests that spread over nearly one-third of the land surface on the earth are the largest reservoirs of biodiversity, carbon sinks, and renewable biological resources. These critical ecosystems provide immeasurable benefits to humanity while maintaining ecological balance and are considered as lungs of the earth by absorbing carbon dioxide and giving out Oxygen [1]. According to Food and Agriculture Organizatio (FAO) (2020) [2], the extent of forests globally is around 4.06 billion hectares, which is roughly a third of the planet′s total surface area. Over half of the world′s forests are situated in just five countries: Russia, Brazil, Canada, the United States, and China (Figure 1). A total of 93% of global forest cover is represented by natural regenerating forests, while the remaining 7% is planted. Since 1990, the natural forest area has decreased (although the rate of decrease is diminishing), and planted forests have expanded by 123 million hectares.

Crucially, forests contain 80% of terrestrial species, including plants, animals, insects, and microorganisms. They provide essential habitat, nutritional resources, and reproductive grounds for countless organisms, many of which remain undiscovered [3]. However, deforestation threatens this biodiversity, leading to species extinction and ecosystem collapse [4]. Trees influence local and global climates by regulating temperatures, maintaining rainfall patterns, and preventing desertification, hence regulating the overall climate [5]. Additionally, trees help with soil protection and water cycle maintenance by preventing soil erosion, enhancing water infiltration, and regulating water flow. Growing interest exists in utilizing trees with short-rotation coppice and high growth rate as second-generation bioenergy feedstocks [6]. Nevertheless, escalating demand for forest products has led to accelerated deforestation. This has created an urgent need for reforestation efforts and the development of dedicated plantations [7]. Traditional silviculture and breeding methods are no longer adequate to address these demands, due to prolonged juvenile growth periods and the lack of native plant genes required to introduce commercially valuable traits [8]. Traditional tree breeding methods, while foundational to forest improvement, are constrained by long generation cycles, limited genetic diversity, and the difficulty of introgressing complex traits such as stress resilience and optimized wood properties [8,9]. These limitations hinder the rapid development of tree varieties suited to contemporary environmental and industrial needs [10].

In response, genetic engineering has emerged as a transformative tool for accelerating tree improvement. Early transgenic approaches enabled the introduction of exogenous genes for traits such as insect resistance and herbicide tolerance, with *Populus* spp. and *Eucalyptus* spp. serving as a pioneer species [11,12]. More recently, the advent of precision genome editing technologies, particularly CRISPR-Cas9, has revolutionized the field by enabling targeted modifications without the integration of foreign DNA [13]. These advances are complemented by multi-omics approaches, which provide systemic insights into gene function and regulatory networks, facilitating the molecular design of trees with enhanced performance [14].

This review presents advancements and prospective trajectories in tree genetic modifications, emphasizing breakthrough technologies, applied methodologies, and the inherent challenges confronting this evolving domain. This review also compiles recent advancements in genetically modified trees (GMTs), focusing on improvements in wood quality, growth, resilience, and their role in ecosystem services. It explores cutting-edge technologies like CRISPR-Cas9 and multi-omics, which are transforming tree biotechnology. While significant progress has been made with trees like poplar, eucalyptus, and *Pinus* spp., this review critically examines the hurdles of implementation, including technical, regulatory, and public acceptance challenges. By integrating technological progress with risk governance perspectives, this work aims to provide a comprehensive roadmap for the responsible deployment of GMTs in sustainable forestry and global climate mitigation efforts.

## 2. Challenges and Opportunities of Traditional Breeding

Traditional tree breeding methods involve the utilization of the existing genetic variability among trees without applying external means to alter the germplasm of the trees for modification [8,15]. Trees are modified through their natural genetic evolution and by the use of traditional techniques, which involve the introduction of new plant species, selective breeding, hybridization, progeny testing, and clonal propagation [9], as shown in Table 1.

Traditional breeding methods have laid a foundation for improved tree varieties that contribute greatly to sustainable forest management, and due to the fact that the desired traits are acquired naturally without the external alterations using technological tools [9]. However, this method takes a very long time to show the results, given that the trees’ growth duration is also long. Moreover, the process of selection is mainly by external features, which might be different from what is desired from the inside of the tree [9,10]. The tree phenotype might tell a little about the desired genotype. For thousands of years, farmers have carefully chosen the best plants to cultivate, improving qualities like yield, taste, and resistance to diseases [16]. Through cross-pollination, breeders merge beneficial traits from different varieties, fostering genetic diversity. However, this method demands patience, as it takes multiple generations, ample space, and years from seven to ten years to establish a stable new variety, which is a long duration for one improvement [15,17]. This is a technique used to introduce uncontrolled genetic mutations into an organism’s DNA. It is commonly used in genetic research, biotechnology, and directed evolution to create genetic diversity and study gene functions. The lengthy generation cycles and intricate genomes of forest trees can hinder the effectiveness of selective breeding. Moreover, environmental conditions may impact phenotypic traits, reducing precision [9]. To ensure trees thrive in their natural habitat, selective breeding must be carried out within designated seed zones, following the principle of sowing the right seed for the right place [17]. Interestingly, traditional tree breeding is still used in many parts of the world to produce improved seeds, despite the increasing availability of biotechnology tools for accelerated improvements [18]. Traditional breeding remains a cornerstone of forest tree improvement in many parts of the world, including Europe. For instance, intensive breeding programs for poplars (*Populus* spp.), particularly through controlled hybridization and clonal selection, have been successfully implemented across several European countries. These programs have developed commercially valuable clones that combine traits such as fast growth, desirable stem form, and adaptability to local conditions, demonstrating the ongoing relevance and effectiveness of conventional methods [18,19].

## 3. Advancements in Molecular Design Breeding

### 3.1. Genetic Engineering Techniques

Genetic engineering has transformed breeding by allowing precise modifications to an organism’s DNA. Unlike traditional breeding, advances in biotechnology enable scientists to directly insert genes from different species into plants or animals, producing genetically modified organisms (GMOs) [20]. Genetically modified trees are those trees whose genomes are being modified by biotechnology tools in order to meet the desired traits [21,22]. Genetic engineering has improved tree traits, with *Populus* spp. modified for easier lignin processing and *Eucalyptus* spp. approved in Brazil for faster growth and higher wood density [23]. Genetic modification is becoming a key strategy in forestry, helping trees adapt to climate change, boosting productivity, and strengthening resistance to environmental stress [14]. The genetic enhancement of forest tree species has progressed from traditional breeding methodologies to advanced genomic and biotechnological innovations [24]. Notably, China has successfully genetically optimized over 100 key afforestation tree species, achieving remarkable improvements in timber growth rates and fruit yield efficiencies through superior genetic cultivars [25]. On a global scale, research initiatives on GMTs have diversified, targeting objectives ranging from improved wood structural integrity to applications in environmental bioremediation [26]. The following section lists and describes in detail some common transgenic techniques in genetic engineering.

### 3.2. Methods of Genetic Engineering

**Figure 2:**
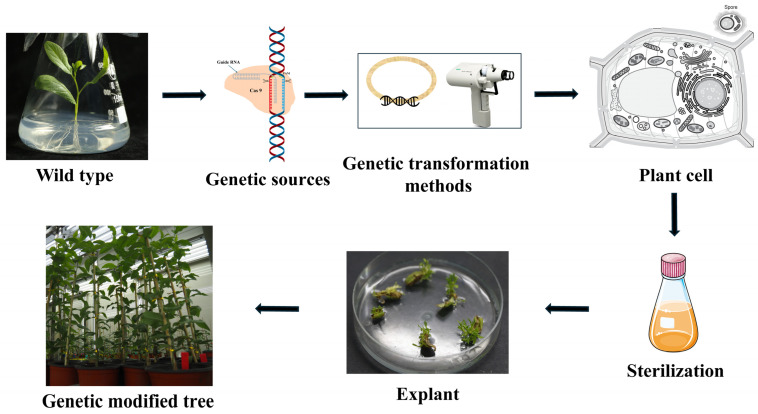
Genetic transformation of trees.

Genetic sources can be isolated from the desired organisms, such as the *Bt* gene, which is obtained from the bacteria [12] and by using biotechnology tools, the gene is inserted into the explant to transform it, and the result is a transgenic plant [27]. This was the traditional method of modifying organisms. It has been receiving opposition from the users who worry about the safety of the introduced genes to the health and the environment [28]. Recent advancements have reduced the worries of the users by coming up with tools that modify the plants and organisms as desired, without adding exogenous genes [29]. Such technologies include mutagenesis, epigenetic modification, and CRISPR-based gene editing. After genetic sources for modification, the choice of an appropriate genetic transformation method is an important step to ensure effective transfections [30]. There are two main types of genetic transformation, namely natural and artificial transformation. In natural genetic transformation, the most preferred is the use of *Agrobacterium*, and in artificial transfer, there is electroporation, gene gun, micro and macro injection, and many others [20]. Each of the methods is effective depending on the wood species, genetic sources to be delivered, and the explant to be used. The most common methods for genetic transformation are the *Agrobacterium*-mediated method and the gene gun method [14]. Figure 2 shows the simplified transgenic operation process for trees.

#### 3.2.1. *Agrobacterium*-Mediated Transformation

The *Agrobacterium*-mediated transformation is the most applied method in plant genetic modification [20] due to its simplicity and efficiency. *Agrobacterium* has a T-DNA region that has growth-synthesizing genes such as cytokinin and auxin. When a plant is wounded, it releases the phenolic compounds that attract the *Agrobacterium* tumefaciens to come and feed on the wounded part. When in contact with the tree, *Agrobacterium* releases its T-DNA region to the plant, which leads to uncontrolled cell division and forms galls on the wounded part [31]. This has been advantageous to researchers by utilizing the same mechanism used by *Agrobacterium* to transfer target genes to the plants by replacing the T-DNA region with the target gene to be delivered to the plant. Zhao et al. obtained *PuZFP1* transgenic lines using *Agrobacterium*-mediated transformation, thereby elucidating the roles of *PuWRKY46* and *PuEGR1* under the transcriptional regulation of PuZFP1 in *Populus ussuriensis* Kom. in the development of lateral roots (LRs) and adventitious roots (ARs) mediated by the ABA/auxin ratio [32]. Recent studies have successfully established an efficient genetic transformation system for eight economically and ecologically significant poplar species (including *Populus trichocarpa* Torr. & Gray, *P*. *ussuriensis*, *Populus alba* var. *pyramidalis*, *Populus alba* × *P. glandulosa*, *Populus deltoides* Marshall × *P.euramericana* cv ‘Nanlin895’, *Populus davidiana* Dode × *P. alba* var. *pyramidalis*, *Populus alba* ‘Berolinensis’) by combining *Rhizobium rhizogenes*-mediated transformation with fluorescent marker-based screening techniques [33].

#### 3.2.2. Biolistic Genetic Transformation Method

It was first introduced by Klein in 1987 [20,34,35]. This is an artificial and indirect method of gene transfer that uses ultrasound to coat the tungsten particles with exogenous DNA and drive them using a gun by high-pressure helium pulses to the tree cells [34,36]. This method is commonly used for monocots and species unable to transform through *Agrobacterium*-mediated transformation. Despite the capability of transferring genes directly to intact tissue, which makes any tissue can be used, the challenge is that the target integrated through this technique is normally present in multiple copies, which can lead to abnormal gene expression and potential co-suppression [20]. Arokiaraj et al. were the first to successfully obtain transgenic rubber trees by transforming anther-derived dedifferentiated calluses of *Hevea* using the gene gun method [37]. Noel et al. introduced the endochitinase gene (*ech42*) from Trichoderma harzianum into black spruce (*Picea mariana* Britton, Sterns & Poggenb.) and hybrid poplar (*Populus nigra* L. × *P. maximowiczii* A. Henry) using the gene gun technique. This resulted in the generation of 15 transgenic black spruce lines and six poplar lines, demonstrating enhanced resistance to fungal pathogens in the trees [38].

### 3.3. Marker Assisted Selection

Molecular markers have advanced marker-assisted selection, making it a powerful tool for rapidly enhancing desirable plant traits [39]. Markers that are closely associated with genes controlling a desired trait can be used to identify and select specific alleles linked with that characteristic. This is a more advanced method of traditional tree breeding, whereby tree breeders use genetic markers to identify and select trees with desirable traits. Instead of relying solely on physical characteristics as in traditional breeding, this allows tree breeders to make precise selections based on DNA markers linked to important traits like disease resistance, drought tolerance, and yield improvement. A DNA marker is a specific sequence of DNA that is used to identify genetic differences between species. These markers help scientists track inheritance patterns, study genetic traits, and assist in breeding programs. This allows scientists to accurately select trees with desired traits by having the information not only on the phenotype but also on the genotype. Marker-assisted selection (MAS) is highly effective for relatively simple traits controlled by a small number of genes [24]. Researchers identify particular molecular markers, such as SNPs or SSRs, that are reliably associated with desirable characteristics. DNA is gathered from every individual and analyzed through methods such as PCR to determine the presence or absence of the desired markers. Research has constructed a complete genetic map of an individual loblolly pine (*Pinus taeda* L.) using amplified fragment length polymorphism (AFLP) markers segregating in haploid megagametophytes and PGRI mapping software ( version 1.0 ) [40]. Another research has used AFLP markers in a two-way pseudo-testcross strategy to generate genetic maps of two clones of different eucalyptus species (*Eucalyptus tereticornis* Sm. and *Eucalyptus globulus* Labill.). The ones showing desired combinations of markers are chosen for breeding, even before those traits are physically evident. These chosen individuals are bred, and their offspring are assessed to ensure they carry the desired genetic markers and display the associated traits. To ensure a successful Marker-Assisted Selection (MAS) program, it is essential to have dependable genetic markers, a robust DNA extraction technique, accurate genetic mapping, clear insight into marker–trait relationships, fast and effective data processing, and access to high-throughput marker detection technologies.

MAS enhances genetic diversity through directed selection by allowing plant breeders to screen and choose individual genetic markers that are linked with desired traits such as drought tolerance or disease resistance, regardless of the phenotypic expressions. This specificity allows low-frequency alleles to be maintained and the genetic diversity to be conserved in the population being bred [41]. For example, in red mangrove conservation, genomic studies have also identified adaptive traits significant to survival in tidal and hypersaline habitats. MAS can spread such traits while avoiding genetic bottlenecks [42]. Moreover, through mapping quantitative trait loci (QTLs), MAS identifies genes for adaptive traits such as tolerance to salt in semi-mangrove species like *Hibiscus tiliaceus* (L.) Fryxell. Such data are utilized in the conservation of climate change-threatened populations [43]. Researchers conducted QTL analysis on a tetraploid F2 willow pedigree at different cold acclimation time points. This pedigree was derived from a cross between a diploid female clone with low cold hardiness and a hexaploid male clone with high cold hardiness, leading to the identification of QTLs influencing fall freezing tolerance and phenological traits [44]. Although QTL maps provide valuable insights, they are expensive and challenging to generate, with their usefulness mostly confined to the specific pedigrees for which they were developed. Despite the shortcomings linked with MAS, the drawbacks of Conventional breeding have been resolved.

### 3.4. Gene Editing Technology CRISPR

This method has been successfully used to boost plant resilience against abiotic stress factors like drought and extreme temperatures [45]. The main advantage of this technology is that there is no external gene that is added to the plant to be modified; it only modifies the plant’s own DNA to achieve the intended trait [46]. Unlike gene editing, the transgenes is performed by inserting other genes from external organisms, which can be a plant, animal, or bacterium. This transfer of genes from different organisms is what makes the GMOs lack public acceptance, as it worries about the user’s health [47].

**Figure 3:**
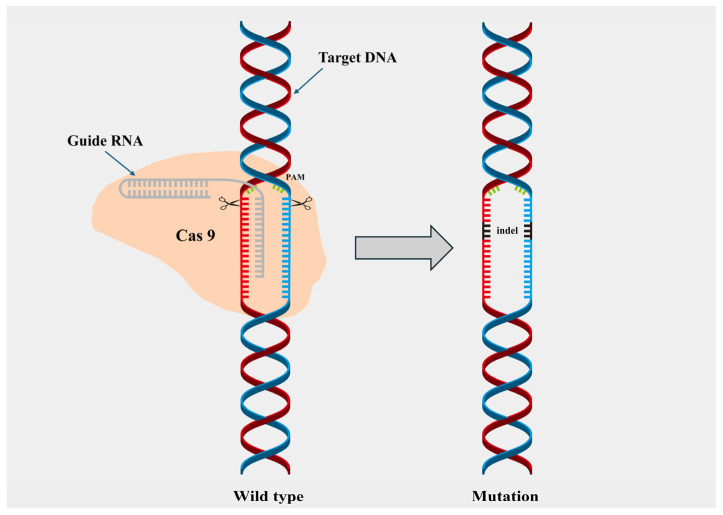
CRISPR-Cas 9 system.

The system operates through two essential components: a guide RNA (gRNA) and the Cas9 protein. The guide RNA has a Cas 9 enzyme, which acts as a pair of scissors for cleaving the DNA segment as required. After the cleavage of the targeted sequence then the cell repairs the part by joining the ends, which is regarded as a indel as shown in Figure 3, or the scientists can then insert another fragment [48].

### 3.5. Applications of CRISPR Genome-Editing

CRISPR technology has revolutionized genetic modification and tree improvement by precisely editing the required modifications. Table 2 demonstrates the application of CRISPR technology in gene editing across different poplar species. CRISPR technology can delete or add the required sequences to achieve the intended modification. Through CRISPR/Cas9, Caffeoyl shikimate esterase (CSE), a central enzyme in lignin biosynthesis, is accurately edited to enhance biomass quality in hybrid poplars (*Populus alba* × *P. glandulosa*), which can be converted into biofuels. In hybrid poplars, which carry four versions of the CSE gene, researchers used CRISPR/Cas9 to selectively deactivate individual genes. Trees with reduced lignin levels up to nearly 30% without any noticeable impact on growth or appearance, even after multiple seasons in outdoor trials. These modified trees also showed a marked increase in sugar release efficiency, making them more suitable for biofuel production. By fine-tuning lignin biosynthesis through targeted gene editing, this study opens the door to creating high-yield, low-recalcitrance bioenergy trees that do not sacrifice field performance [13]. Photoperiod is a key environmental signal that influences seasonal behaviors like growth cessation and winter dormancy in perennial woody plants. In poplar, two photoperiod-related modules, which include *LHY2* and GIGANTEA-like genes, regulate the dormancy-associated gene *FT2*. However, altering *LHY2* and GIs alone does not fully prevent growth cessation under short-day conditions, suggesting other factors are involved. One factor is *PtoHY5a*, which directly activates *FT2* and indirectly enhances its expression by repressing *LHY2*. *PtoHY5a* suppresses short-day-induced dormancy and bud set, while its knockout promotes dormancy. It also inhibits bud-break by regulating gibberellic acid levels and acts downstream of *PHYB2* to control seasonal growth [49]. Loss of function of *PtoHY5a* promotes premature growth cessation and bud set under short daylength. This is helpful for the modification of trees grown in cold climates and in winter periods to make them more resilient and sustain harsh conditions. Albino phenotypes appeared in hybrid poplar plants when both alleles of the *PagPDS1* gene were edited by CRISPR/Cas9, confirming the efficiency of this genome-editing system. Furthermore, no off-target alteration was detected in the closely related *PagPDS2* gene, although it also contains a similar target sequence, showing the high specificity of the designed sgRNA. These findings show that CRISPR/Cas9 can be employed to make precise and efficient targeted genetic changes in trees, offering an excellent tool to enhance favorable traits such as biomass production and stress tolerance for future tree breeding programs [50]. Abiotic stress has been a problem for trees and specifically drought and salt stress. Tree mortality is higher in dry areas and especially when the changing weather is prolonged. There is a need to develop a defense mechanism for the trees to withstand these conditions. CRISPR is the most precise one for targeted modification. The study on *PagHyPRP1* in poplars has revealed its roles in salt and drought stress tolerance. The overexpression lines, knockout mutants, and wild type were treated with PEG-6000 and NaCl, and the results showed significantly lower growth of the wild type and overexpression lines with wilting and withering, while the knockout mutants maintained a normal growth with higher height, root dry weight, stem diameter, and stem dry weight and root shoot ratio [51]. On the other side, *PtoMYB142* has been studied and found to positively regulate drought by activating the gibberellin breakdown gene, which, upon amplification, reduces levels of gibberellin and hence growth is reduced to allow the trees to conserve energy and resources for the time being. The experimental setup involved the wild type, overexpressing the gene, and knocking out the gene. After drought treatment in the soil, the overexpression lines showed diminished leaf size, the reduction in vessel lumen area, and vessel density in the xylem due to less gibberellin. But upon external application of gibberellin, the seedlings maintained healthy growth. On the other side, the wild type and knockout plants suffered severely from the drought treatment, and even after rewatering, the Cas9 seedlings still remained dry [52].

Additionally, CRISPR is utilized in biotic stress resistance in trees by activating pathogen resistance genes or switching off disease-susceptible genes in the tree’s genome. Two transcription factors have been studied for their function in pathogen resistance in *Populus tomesa*, *PtoWRKY18* and *PtoWRKY35*. When subjected to pathogen melampsora, the wild-type seedlings and the knockout mutants were shown to be affected by the treatment, while the overexpression lines showed no sign of being affected, and their levels of pathogen resistance genes were upregulated upon being subjected to pathogen melampsora [54]. In the hybrid poplar 84K (*Populus alba* × *P. glandulosa*), *PagGLR2.8* is a glutamate receptor gene that regulates vascular development. In order to study its function in fiber biosynthesis and composite performance, this gene was knocked out, and the results showed that loss of function PagGLR2.8 resulted in greater stem diameter with about 39.1%, 40.2, and 36.4% greater xylem section on the knocked lines than the control. Higher cellulose content provides the tree with rigidity and protection against pests and diseases [55]. Knock-out lines in this study increased cellulose content by 5.9%, 9.8% and 2.6% greater than the control, and more cellulose was located more on the secondary cell wall in the CRISPR-edited than in the control [55]. Lignin is important for plant growth and support; however, higher lignin content does not favor industrial processing of wood [57]. Thus, wood with less lignin content is good for the easy processing of manufacturing wood products [58]. It is revealed that knocking out *PagGLR2.8* decreased the lignin content by 26.3%, 23.5% and 22.7% in knockout lines 8, 9, and 17, respectively. Additionally, knockout had also longer phloem and xylem fiber length than the control plants. On the transcriptome analysis through RNA-seq showed than many differentially expressed genes were downregulated than those which were upregulated and all these downregulated genes are involved in wood formation. Crispr editing of *PagGLR2.8* proved to be effective for the modification of lignin, cellulose, and phloem fibers for making high-performance composites [55]. Male sterility (pollen-free traits) is highly valuable for human health, economic benefits, biodiversity conservation, and agricultural applications. For instance, pollen-producing plants like sugi, cypress, birch, and certain pasture grasses release large amounts of allergenic pollen, contributing to widespread pollen allergies. *CjACOS5* is involved in the production of pollen in sugi. Since the produced pollen has been a long-time problem for the people, researchers have dived into finding a solution to this problem. By developing sterile varieties that do not produce pollen, we could significantly reduce the number of people suffering from pollen-related allergies. In *Cryptomeria japonica*, *CjACOS5* has been knocked out in order to study its function, and the results showed its disruption is useful for the generation of pollen-free sugi. CRISPR has revolutionized tree breeding technology by correcting many difficulties that were in traditional methods of tree breeding [56]. This gene editing technology has high precision editing and provides many options for genome editing without altering the original DNA sequence. Despite its strength in the field of biotechnology, especially in trees, there are some off-target events happening while editing genes, and these mutations cause undesired changes that might be harmful if inherited. Trees have a complex and large genome of trees that makes it hard to identify it all. 

**Figure 4:**
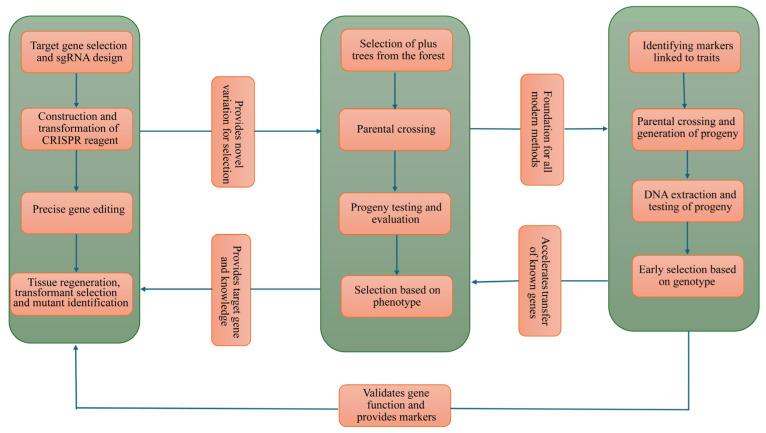
Interaction between traditional breeding, Marker Assisted Selection, and the CRISPR editing method.

Despite many of the revolutions brought in biotechnology by the CRISPR method in trees, its performance is downgraded by a number of issues. Transformation efficiency of the gene-edited lines is generally low, as it has been demonstrated that the transformation efficiency of Hbpds, a Phytoene desaturase gene, was 3.2% with the CRISPR vector [59]. Another challenge with CRISPR is the sgRNA design, which crafts the RNA sequence that guides the Cas9 protein to the specific target. Good design ensures high editing efficiency and low off-target effects. SgRNA design has greatly affected the editing of transgenic poplar, which had a 95.5% mutation on both gene alleles. On the other hand, a mutation on both alleles of 20.8% on *CSE1*-sg2 lines was observed with high chimeric and monoallelic mutation [13]. This shows how the sgRNA design can affect the resulting mutation. The large and complex genome sizes of tree species have led to the availability of very few complete genome data of tree species. Therefore, for trees with no genome data, we use reference genome data, which are available in different sites. The design of target-specific sgRNAs is very important for efficient editing of the target gene [60,61]. However, reference genome data might not be a perfect match for all trees, making it hard to predict target and off-target mutations [61]. However, with advancements in biotechnology, the number of fully sequenced tree genomes is increasing [62]. Figure 4 illustrates the interactions among traditional breeding, Marker Assisted Selection, and CRISPR editing methods. Through the integration of these multiple technologies, we aim to develop a more accurate, efficient, and practical engineered breeding system.

**Figure 5:**
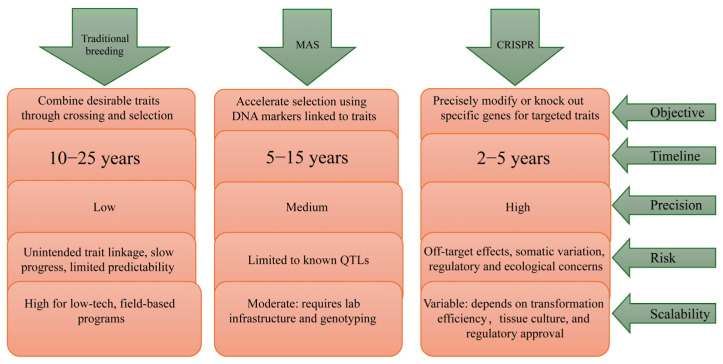
Parameters of traditional tree breeding, MAS, and CRISPR.

Traditional breeding acts as a backbone for all the advancements in biotechnology, as it provides foundational genetic crosses and phenotypic genetic crosses and phenotypic data is required to identify important traits that need further validation and improvement. CRISPR acts as a powerful tool for creating genetic diversification by creating precise variations in genes that control important traits such as disease and insect resistance, cold and drought tolerance, in a single generation. Thus, CRISPR serves as a powerful tool for creating novel and improved lines that are used as markers and provide reference lines and excellent parents to be used in traditional breeding programs. Additionally, CRISPR creates new and valuable alleles that breeders can use for selection. As the new lines have been created, MAS acts as a tool for monitoring the marker in the coming generations and selecting accordingly. Through MAS, traditional breeding becomes more efficient by allowing breeders to select trees based on their genotype, not only phenotype, but also in early years. Figure 5 illustrates the differences among the three breeding methods.

## 4. Applications of Genetic Modification in Trees

### 4.1. Wood Qualities Improvement

Lignin acts as a surface where enzymes irreversibly bind in a non-productive manner, significantly reducing the efficiency of enzyme-driven hydrolysis. Removing lignin from wood is challenging due to its complex chemical structure, which binds tightly to cellulose fibers, requiring harsh processing conditions with high temperatures and strong chemicals to remove it and access other cell wall contents such as cellulose and hemicellulose [63]. It has been demonstrated that the greater the number of pores created by lignin removal, the more favorable the environment for the enzymatic activities of hydrolysis. This process is energy-intensive, driving up operational costs, while also producing significant waste and pollutants that need further treatment to minimize environmental harm [58]. In addition, while lignin can potentially be utilized in biofuels and chemicals [64]. To date, technology has been unable to cost-effectively convert it into useful products, reducing economic incentive for its removal [65]. Scientists are attempting enzyme-based and biocatalytic methods in an attempt to make the process cost-friendly and eco-friendly [57]. Genetic engineering has overcome this problem by modifying the DNA of the tree in such a way as to lower the lignin content [23] or make the depolymerization of lignin easier without compromising the environment too much [57] for paper and pulp producers such as *Populus* spp. and *Eucalyptus* spp. Scientists have identified a number of problems associated with the alteration of the lignin content of wood [66]. The alteration that is achieved is the downregulation of genes, which results in the production of lignin in wood, or the inclusion of genes, which will make lignin more hydrolysable during hydrolysis [23,57,65]. Hybrid poplars (*Populus alba* × *P. grandidentata* cv. Crandon) were genetically engineered to introduce ester bonds into the lignin polymer backbone through the expression of the *Angelica sinensis* gene. This was successfully achieved in hybrid poplar (*Populus alba* × *P. grandidentata*) by using both a universal 35S promoter and the poplar *CesA8* xylem-specific promoter involved in secondary cell wall cellulose biosynthesis [67]. The modification improved saccharification efficiency under various pretreatment conditions and kraft pulping performance relative to wild-type poplars [23]. It has been emphasized that downregulation of the genes and transcription factors participating in lignin polymer biosynthesis enhances wood processing efficiency and saccharification efficiency. Researchers used predictive modeling to set goals for reducing lignin levels in poplar trees, increasing the carbohydrate-to-lignin (C/L) ratio, and raising the ratio of two key lignin monomers—syringyl (S) and guaiacyl (G)—known as the S/G ratio. These combined chemical traits represent the chemical sweet spot for fiber production. Based on these models, the authors selected seven optimal strategies and used CRISPR gene editing technology to develop 174 poplar lines. The results showed that some varieties achieved up to a 50% reduction in lignin content, while others saw a 228% increase in the C/L ratio [68]. The less the lignin content, the higher the efficiency and smooth processing of wood products, and fewer chemicals are required to process the wood. Additionally, wood processing from wood with less lignin is environmentally friendly.

Despite the economic value of lignin-engineered trees, however, low lignin in a wood also has negative impacts on the tree. Lignin modification in trees by downregulating genes such as *CCR*, *CAD*, or *COMT* can greatly enhance the processability of wood for biofuels and pulping by decreasing lignin content or by changing the composition of lignin [23]. Most such modifications are, however, followed by trade-offs that include decreased growth, xylem integrity, susceptibility to drought and pathogens, and cytotoxic phenolics accumulation as a result of metabolic rerouting [69]. Silencing lignin biosynthesis genes such as *CCR* in poplars causes an increase in various extractable phenolic compounds in xylem, compositional alteration of the cell wall, which can lead to bacterial colonization as the cell wall is damaged, but also a reduction in tree strength and mortality [70]. About a 50% decrease in growth was observed for downregulated poplars with the *CCR* gene for 160% improvement in ethanol production [23]. Multi-year field trials have demonstrated that although some of the designed lines have good performance under controlled conditions, their performance in the field is variable, even negative [70]. Such trade-offs emphasize the need for balanced approaches that optimize lignin traits without compromising tree vigor or environmental resilience.

### 4.2. Drought Tolerance

**Figure 6:**
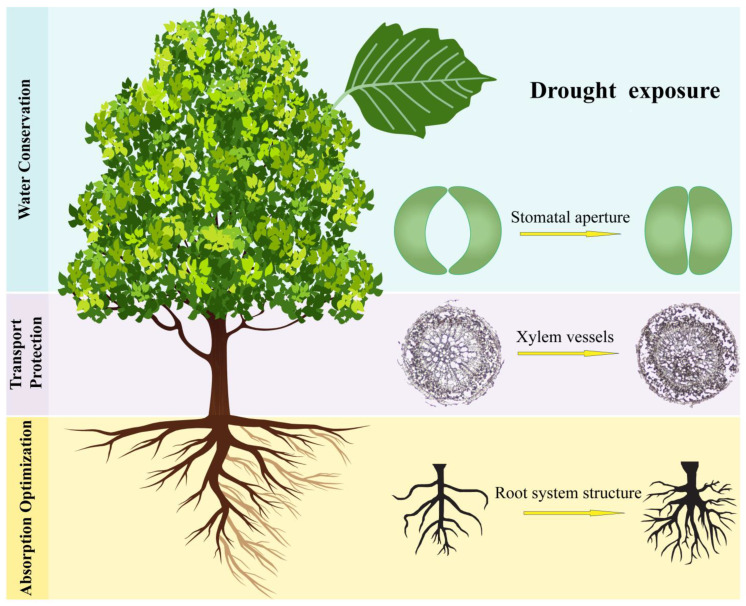
Drought response mechanisms in forest trees.

Drought is one of the abiotic stresses that affects most trees due to the climatic changes [71,72,73]. It is the period of prolonged water deficit in plants, such that the plant’s rate of transpiration is becoming higher than the rate of the root’s water uptake, which, when it becomes severe, causes permanent cell damage [74]. In the natural state, plants have mechanisms that enable them to cope with water deficit, which are physiological, biochemical, and molecular responses [75]. However, due to climate change, prolonged periods of drought cause increased tree mortality [76]. Figure 6 shows the response generated when trees are exposed to drought conditions. This drives the need to advance trees’ genomes and proteomes to tolerate harsh changes. Plants’ Abscisic acid functions in regulating growth, development processes in plants, such as seed maturation, germination, flowering, and movement of stomata. Moreover, it reacts to different abiotic stresses such as water deficit [77]. When there is a water shortage in the soil, ABA production rises in the roots and leaves, which then acts as a signal for the downstream pathway. The ABA signal is then perceived by the primary receptors, which attach themselves to the hormone, which then inhibits protein phosphatase 2C, PP2C. protein phosphatase 2C is one of the distinct groups of enzymes that depend on magnesium and manganese ions to function, and plays a key role in stress signaling [78]. PP2C is then inhibited by ABA, and a kinase called sucrose non-fermenting-1-related protein kinase 2 is activated to phosphorylate the downstream targets. These kinases are found in plants and serve as the main controllers of stress response in plants [79]. The SnRK2 then activate the transcription factors such as Abscisic acid insensitive 5, which upon binding to the promoters of the drought responsive genes will influence the expression of the genes responsible for making proteins that will bring responses to drought such as the stomatal closure, production of ROS scavenging molecules, water use efficiency levering, osmotic adjustment through production of many non-armful molecules and ions such as proline, protecting the membranes [80].

Genetic modification of trees for drought tolerance involves altering their DNA to enhance their ability to withstand water scarcity [72,73,81,82,83]. Scientists use various techniques to introduce drought-resistant traits, such as improved water retention, deeper root systems, and enhanced stress response mechanisms [84,85,86]. Genetic modification has enabled drought-susceptible trees to overcome harsh climate changes and periods by increasing the expression of the drought-responsive genes to make the proteins required to bring the response to drought, such as stomatal closure, synthesis of non-harmful solutes to balance the changing osmotic potential [72,73]. Moreover, by enhancing the ability of plants to improve water use efficiency, having long roots enables the trees have the ability to withstand the harsh environment of high temperatures and low water. Additionally, overexpressing genes responsible for producing proteins that function in regulating the plant survival under drought conditions will help the trees survive adverse water deficiency. Researchers successfully created new drought-resistant germplasm in poplar by utilizing and optimizing the CRISPR/Cas9 system to perform multisite precision genome editing on the drought stress response factors *PagHyPRP1*, *PagHyPRP1A*, and *PagHyPRP1B*, which are primarily expressed in the roots of *Populus alba* × *P. glandulosa* [51].

When the roots sense the dry conditions, often there is a rise in Abscisic acid (ABA) on the extracellular and this rise acts as a signal which is transported towards the shoot of the plant, the rise in ABA will lead to high production of the calcium ions inside the cells of the plant and each signal inside the cell is recognized by its specific sensor [87]. These calcium signals relating to drought will be sensed by the calcium sensors responsible for activating the downstream target genes to produce required proteins and bring about required responses [88]. The kinases are responsible for sensing the calcium ion signals, which then phosphorylate the downstream target genes for making proteins that will bring about a response to drought [89]. Genetically modified genes can improve water-use efficiency, optimize root structures for enhanced water uptake, and control stomatal function to minimize water loss [72,82]. Additionally, certain modifications promote Osmo protectant synthesis, helping plants maintain cellular integrity during drought stress [81]. Genetic alterations frequently focus on ABA, ethylene, Jasmonates, and salicylic acid (SA) pathways, all of which are essential for drought resistance [90]. These hormones govern stress signaling, stomatal regulation, and metabolic adaptations, enhancing a plant’s ability to withstand water scarcity [91]. *PeFUS3* is a key regulator, maintaining root growth of poplar by modulating the crosstalk of auxin and ABA signaling under drought stress [92]. Researchers utilize stress-responsive promoters to trigger drought-tolerance genes exclusively under water-deficient conditions. Studies have shown that the expression level of *PdGNC* significantly increases under ABA and dehydration treatments. Further drought stress response analysis revealed that poplar seedlings overexpressing *PdGNC* exhibited reduced leaf stomatal aperture, significantly lower water loss rates compared to control plants, and higher water use efficiency. In contrast, CRISPR/Cas9-mediated gnc poplar mutants displayed enlarged stomatal aperture, accelerated water loss, and reduced drought tolerance [93]. Promoters tend to increase the expression of the candidate genes when the plant is exposed to drought stress [72,74]. This approach minimizes excess energy use and enhances the plant’s ability to endure drought stress efficiently [73]. *Arabidopsis thaliana* (L.) Heynh., a model organism widely used in plant research, was one of the earliest plants genetically engineered for drought tolerance [82]. Moreover, researchers have also modified poplar trees to enhance their ability to withstand drought by altering genes related to water-use efficiency and stress response [21]. The modified trees have an improved performance when it comes to drought stress. Additionally, studies have demonstrated that transgenic *Pinus* spp. with enhanced ABA biosynthesis and stress-response genes exhibit improved drought tolerance [73].

### 4.3. Insect Resistance

Insect-resistant tree species play a crucial role in maintaining forest health by reducing vulnerability to pests and minimizing the need for chemical pesticides [94]. In order to minimize the effects posed by the harmful insects to the trees, breeders have engineered the DNA from a bacterium that has a natural toxin to biodegrading insects [95]. This DNA in an engineered plant produces a protein that is harmful to biodegrading insects but not to human beings. Some trees, like *Bt* poplar (such as *Populus nigra* and various hybrid poplars), have been genetically modified through methods like *Agrobacterium*-mediated transformation and biolistics to withstand insect attacks, while others, such as ginkgo and magnolia, possess natural defenses against pests [12]. Meanwhile, studies have also shown that trees can defend against bark beetles and the fungal pathogens they carry by producing resin. The resin accumulates at the wound site, effectively killing the invaders and sealing the wound [96]. These trees are aiding sustainable forestry, enhancing timber yields, and preserving ecosystems. However, careful management is required to avoid unwanted ecological imbalance, such that the resistance traits do not decrease biodiversity or food chains [97]. China became the first nation to cultivate GMTs commercially when, in 2002, it started releasing two poplar species that are resistant to pests and are still the sole GMTs in large-scale cultivation today [28]. Brazil has approved the commercial use of genetically modified eucalyptus for increased biomass [98].

### 4.4. Phytoremediation

**Figure 7:**
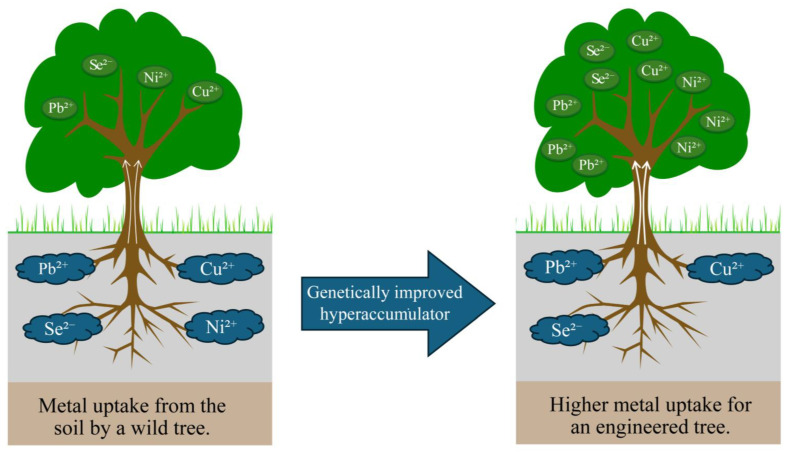
Tree showing accumulated heavy metals absorbed from the roots in its parts.

Phytoremediation is a sustainable and environmentally friendly technique that harnesses plants and their interactions with microbes to eliminate various organic and inorganic contaminants from polluted soils, water, and sediments [99]. There are various mechanisms involved in phytoremediation, such as phytoextraction, Phyto stimulation, Phyto filtration, Phyto stabilization, and phytovolatilization [99,100]. This cost-effective and low-impact method serves as a promising alternative to traditional physical and chemical treatments, which often pose secondary pollution risks [27,101,102]. As a result, phytoremediation is gaining increasing recognition among researchers and broader public support [103]. In the current industrial advancement era, a lot of harmful chemicals and heavy metal pollutants are produced from the industries, which are very harmful to the well-being of the environment in general [104]. In past years, there have been other methods used to do remediation of the soils, which involve using chemicals and physical methods that actually clean the soil and water, but pose other toxins following the method used [105]. To combat this shortcoming, researchers have been successful in identifying plants that have the ability to absorb heavy metals such as mercury (Hg), lead (Pb), copper (Cu), and nickel (Ni) in the roots and store them in the stem and leaves [27,101,104,106]. Figure 7 schematically illustrates the uptake of heavy metals by tree hyperaccumulators. Plants that are able to accumulate heavy metals are called hyperaccumulators. However, the ability to accumulate heavy metals varies significantly between species and among cultivars within species [107]. Research has shown that transferring specialized genes from hyperaccumulators to fast-growing plant species significantly improves their ability to absorb and process contaminants [108]. Advances in transgenic plant development highlight the effectiveness of this method in improving phytoremediation potential [104]. Currently, various biotechnological strategies are being used to remediate heavy metals (HMs) such as cadmium (Cd), Hg, Pb, selenium (Se), and arsenic (As) from polluted environments [27,109]. Genetic engineering enhances plant functions by modifying processes like transport, metal absorption, accumulation, tolerance, chelation, and vacuolar sequestration, unlocking new opportunities for phytoremediation and environmental restoration [27]. Studies in *Populus canescens* (Aiton) Sm. have demonstrated that applying appropriate concentrations of exogenous ABA significantly enhances the translocation of Pb from roots to shoots, resulting in a 39% increase in Pb accumulation in leaves. This process is associated with upregulated expression of lead transport-related genes such as *NRAMP1.4*, *ABCG40*, and *FRD3.1*, which collectively promote the translocation and accumulation of Pb in the above-ground tissues of poplar [110]. Poplar trees have a well-developed root system, exhibit high tolerance to external toxic substances, and possess the capacity to enrich and transport heavy metals to their aboveground parts [111,112]. Therefore, poplars have been widely used to remediate heavy-metal-contaminated soils in North America [113].

### 4.5. Carbon Sequestration and Climate Change Mitigation

Carbon dioxide sequestration refers to the act of absorbing carbon dioxide from the air and storing it in various forms. When plants are involved in capturing carbon dioxide, it is biological carbon sequestration [114]. Plants use the carbon dioxide from the air to make their own food through the process of photosynthesis. Around 50% of a tree′s dry biomass consists of carbon. because they can store carbon as biomass for the entirety of their life cycle [115]. However, the rate of carbon dioxide emission is abruptly increasing from industrial advancements. This leads to glacier melting, rising sea levels, increasing ocean acidity, and unexpected climate shifts [114]. Through genetic engineering, plants are improved for photosynthetic efficiency by absorbing excess carbon dioxide from the air and using it for photosynthesis and by storing excess in their biomass. For example, studies on transgenic poplar with modified lignin content have shown not only improved saccharification efficiency but also increased biomass yield, contributing to greater carbon sequestration [116]. Similarly, fast-growing GM pine varieties have demonstrated potential for enhanced carbon uptake in plantation settings [117]. Utilizing fast-growing species for carbon sequestration is a great strategy for meeting the global demand for wood products while fighting climate change.

### 4.6. Biodiversity Restoration

Genetic engineering can be applied to wild-type trees, hybrids, and clonal materials to enhance resilience to environmental stresses. Biocontrol strategies use engineered organisms to manage invasive species that threaten ecosystems. Notably, the uniqueness that native species bring to the specific location; however, the rapid climate changes and invasive species make them unable to sustain severe changes in their environment. Therefore, genetic engineering helps the native species by altering their DNA to be more resilient to climate change and have stability to withstand the invasive species. GMTs are increasingly being explored for their potential in forestry, environmental conservation, and commercial applications, as stated above. Various species have been engineered for a specific purpose and have mostly shown positive results. The use of GMTs in biodiversity restoration raises important ecological and ethical questions. While GMTs can be engineered for enhanced stress tolerance and growth, their introduction into natural ecosystems may alter species interactions, evolutionary trajectories, and ecosystem dynamics. For instance, gene flow to wild relatives could affect genetic integrity, and trait changes may influence associated flora and fauna. Therefore, the deployment of GMTs in restoration must be guided by robust ecological risk assessments and long-term monitoring [118]. The question of whether GMTs can be considered ‘natural’ also touches on deeper philosophical and societal values, underscoring the need for inclusive dialog among scientists, policymakers, and the public.

### 4.7. Herbicide Tolerance

Weeds have significant negative effects on plant and tree growth by competing for essential resources such as water, nutrients, sunlight, and space. This competition can lead to stunted growth, reduced yields, and weakened resistance to pests and diseases. Trees have been genetically engineered to resist herbicides such as glyphosate and glufosinate. GMTs have been developed to exhibit herbicide tolerance, allowing them to withstand specific herbicides while controlling unwanted vegetation. The hybrid poplar clone (*Populus alba* × *P. grandidentat*) was genetically engineered for glyphosate tolerance using *Agrobacterium*-mediated transformation [119]. Suzano and its subsidiary FuturaGene have engineered genetically modified eucalyptus varieties resistant to glyphosate. These modifications help prevent yield losses while allowing for uniform, mechanized herbicide application using tractor-mounted sprayers, reducing chemical waste and lowering operational costs [11].

## 5. Current Status of Genetically Modified Trees

### 5.1. Progress in Research and Development and Field Trials

Although the development of GMTs has historically lagged behind that of GM crops due to their longer life cycles and ecological considerations, significant progress has been made in enhancing key traits such as growth rate, insect resistance, and wood quality [6]. Notably, China has emerged as a pioneer in the commercial application of GMTs, with several insect-resistant *Populus* spp. varieties being widely cultivated [120]. However, environmental concerns, regulatory challenges, and public perception continue to shape the future of GMTs. As of 2019, a total of 225 field trials of GM trees in 16 countries have been recorded in the field trial database of the Food and Agriculture Organization (FAO, 2020) [2]. The majority of these trials were conducted in North America and Europe, followed by Asia. A limited number of trials have been reported in South America and Africa. Despite significant progress, tree genetic modification faces many technical challenges. Long life cycles of trees make it hard to precisely transform trees [21,121,122]. The assessment is performed only based on the initial performance. Subsequently, some of the experiments give good results when tested in the controlled environment; however, they deviate when exposed to the field trials. As a result, slows down the research progress on trees compared to annual crops. Furthermore, there are many ongoing research studies conducted by universities, institutions, and companies for various modifications to be performed in trees. Despite some of the modified trees being approved for commercial release, deployment of many species becomes difficult due to the opposition from the local communities, government regulations, failure to pass the assessment procedures, and additionally, some modified trees show no significant benefits when compared to the non-modified ones. In Europe, field trials of GMTs are largely prohibited due to stringent regulatory frameworks and public opposition. Table 3 shows different species and companies, and universities performing field trials for a number of species for various traits. Amongst all, China is the leading country for performing research and performing field trials, followed by Brazil and the United States of America while research studies indicate that there are no field trials in Africa [28].

### 5.2. Threats Posed by Genetically Modified Trees to Biodiversity Loss

With extended life cycles of trees, predicting their future interactions with ecosystems and other species remains challenging. Significant concerns have been expressed about introducing transgenic trees into natural ecosystems, particularly regarding the long-term stability of transferred genes in long-lived tree species and the potential spread of recombinant DNA through various environmental pathways. When genes or organisms spread beyond plantation limits, they pose uncontrollable risks to surrounding ecosystems, potentially leading to irreversible consequences, similar to other introductions of mobile biological entities. More than half of the surveyed researchers have discovered the escape of GM pollen and plants into native ecosystems and forests, which raises concerns about the contamination of native species with engineered organisms. Once the engineered species are deployed in the plantations, pollen and seed flow to non-target organisms is inevitable [128]. The concern is that upon acquiring the transgene for resistance to different stresses, pests, and diseases, some wild species might become aggressive invaders of natural areas and farms [128]. According to current research on genetic modification, genetically improved trees with traits such as insect resistance and herbicide tolerance may disperse pollen over distances of several kilometers. The potential invasion of transgenic tree species could threaten the local ecological balance [129]. Honey contamination with genetically engineered (GE) pollen is unavoidable, as approximately 35% of Brazil’s honey production originates from eucalyptus. The country is home to 350,000 honey producers [28]. However, research efforts have shed light on this by developing containment methods in different forms that use genetic modification to avoid the escape of engineered genes from the transgene trees to non-targets through inducing sterility or by removing the transgene from gametes before deploying them [130]. Mitigation procedures intend to reduce the fitness of transgenes by tightly linking them to an engineered gene that is maladaptive in the wild, hence providing a useful complement to the expected leakages in containment strategies. Additionally, due to the promising results of the genetically engineered trees, native forests are cleared to establish the field trials and plantations. Clearing native trees for establishing plantations and field trials poses biodiversity loss and genetic instability of the trees. Many plantations are monocultures with uniformity, which makes the trees susceptible to insects and diseases, unlike in wild forests. Natural biodiversity should be conserved to preserve genetic biodiversity.

### 5.3. Regulatory and Public Acceptance

The successful endorsement of genetically modified trees does not only depend on the advancement of science and technology but also on how society accepts them. The occurrence of horizontal gene transfer breaks the boundaries of closely related species in forest trees, intensifying the complexity of gene flow. For instance, the expression products of transgenes in genetically modified trees can impact soil ecosystems through tree residues and root exudates [131]. Many fear that GMTs could unintentionally spread modified genes to wild populations through long-distance pollen and seed dispersal, posing risks to biodiversity [132]. Limited but detectable gene flow was detected in a wild Populus individual from insect-resistant *Populus nigra* species field trials, which have been monitored for 12 years [133]. However, the transgene did not persist for long, which suggests low ecological risks for well-managed conditions such as buffer zones [133]. Some communities also oppose GMTs on ethical grounds, consider them as not unnatural, and prefer traditional forestry practices. Concerns extend to their interactions with microbiomes, as GMTs might alter soil and microbial dynamics in unpredictable ways. These uncertainties contribute to cautious regulatory oversight and public resistance to the widespread adoption of GMTs. Likewise, research on poplar trees highlights the intricate interactions between trees and their microbiomes, making it challenging to accurately predict the performance of GMTs across varying environmental conditions [134]. A study on the effect of the presence of transgene on the bacterial abundance was analyzed on the trunk tissue, root tissue and rhizosphere soil of both insect resistant poplars and non-transgenic poplars was analyzed and found not to be significantly different indicating that, the presence of transgene had no effect on the abundance of the bacterial community [135] contrastingly with a study of *PaGLK* transgenic poplar and wild type which had a significant difference in the abundance and diversity of bacterial community on the wild type than the repressed line and higher fungi abundance on repressed expression than the wild type indicating that gene editing have effects on root exudates, rhizosphere soil enzyme activity and soil microbial community [136]. Because biological diversity has multiple levels, from molecular interactions to entire ecosystems, current methods are insufficient for fully evaluating the impact of genetic modifications on wild organisms. In China, despite significant advancements in tree genetic improvement, ongoing efforts are required to enhance seed market oversight and improve information services, indicating that regulatory policies are still evolving [26]. The commercial adoption of GMTs is hindered by regulatory barriers and market restrictions. Many countries enforce strict approval processes, requiring extensive environmental impact assessments before granting commercial approval. Due to concerns about transgene escape, GMT research is often confined to controlled environments, limiting large-scale field trials. Additionally, international regulations vary, with some nations outright banning GMTs, while others proceed cautiously with their deployment. Market acceptance is another obstacle, as certain industries and consumers favor non-GM wood products, restricting trade and commercial viability [121]. Historically, the government regulations were strictly regarding the release of the GM plants to the environment, mainly contributing to the opposition from the local communities; however, recent advancements in producing exogenous free plants and trees have led the government to pull back regulations regarding field testing and commercial release of the GMs to the market [28]. However, this is not good as random release of GMTs to the environment without risk assessment might pose serious problems socially, ecologically, and economically. Despite the many challenges faced by the GMTs, research has been advancing to find solutions to these challenges. Gene flow can be reduced by sterilizing GM trees and transgene mitigation in order to avoid the escape of pollen from GM plantations to the non-GM plantations. Transgene mitigation is a useful strategy to help prevent genetically modified traits from spreading into non-GM plant populations. This method works best when the mitigation gene designed to reduce fitness is strong enough to limit survival or reproduction. However, it has been demonstrated that, if the mitigation gene becomes separated from the main GM trait, it can lead to the rise in new, advantageous traits in conventional plants, which poses a risk. That risk drops significantly if both genes stay closely linked and inherited together [137]. Moreover, to effectively control the spread of GM traits, it is important to keep the mitigation and primary genes tightly connected. Assessing the risk of gene separation requires detailed data on genetics, local plant behaviors, and how GM and non-GM plants move and mix. The ability to generate trees that are unable to reproduce would allow control programs to put more effort into the existing trees and give forest owners freedom to operate for new plantings [138].

### 5.4. Socio-Economic Dimensions Associated with Genetically Modified Trees

#### 5.4.1. Intellectual Property Rights

Currently, there is an increasing innovation and research of forest trees and breeding of improved genetic sources to meet economic, environmental, and ecological aspects. To sustain this, two intellectual property rights must exist to provide equilibrium between the two. Exclusive rights give the inventors and the plant breeders the mandate on their products for the time specified under their agreement [139]. While patent rights protect the innovators of the technologies, tools used, and processes for producing novel plant varieties, plant breeders’ rights protect a plant variety [139]. This helps to create a good harmony among the innovators, breeders, and users. However, it also creates a bias to those who cannot afford to buy improved genetic sources for their plantations, and the risk of pollen transfer from the plantations with improved genetic sources to the wild is not fully controlled [140]. There are many inventions on different traits, genes, and tools for tree improvement, and together with breeding companies, there are many modified tree varieties that are mostly still confined to field trials, while a few have been grown commercially in plantations, such as poplars in China [12]. New companies seek patents to have more investors, as they need to secure the innovations and obtain a return by licensing them; however, managing intellectual property rights is overwhelming due to its complexity and expense, which makes the large companies keep dominating [141]. The plant breeder’s rights are valid for 25 years for trees, and during that period, farmers are allowed to save seeds for the coming planting period. However, in patent time, which is 20 years, third parties are not allowed to use the invention unless they have a license [139,140]. Patents help in protecting the inventors with their inventions, but also promote advancements in biotechnology by attracting more research and development of different plant varieties through breeding. Patents also help in increasing the genetic gain of trees while maintaining genetic diversity. Norway has gotten a solution to the growing wood demand that was not met by the conventional breeding method; however, the terms of the patent holder might conflict with “every man’s right” of the Norwegian public land, and this should be taken into consideration before deploying the seedlings resulting from this somatic embryogenesis patent [140].

#### 5.4.2. Access to Technology

Notwithstanding many hurdles tied to the genetic transformation of forest trees [14], genetic engineering has been able to yield meaningful results that solve the problems linked to conventional breeding through technologies such as CRISPR-CAS9, TALS, and ZFN [6,61,66,72,142,143,144,145,146]. These technologies are mostly developed by companies that protect their inventions through intellectual property rights [139,140,141]. Many companies for tree improvement are allocated in developed countries such as Brazil, the United States, and China. Such companies include Suzano and Arbogen [28]. Given the costs associated with the whole process of inventing and publishing patents [140], small companies and public institutions in developing countries cannot afford the costs to license the patents. Many developing countries lack research funding, trained personnel to develop and adapt GM trees. As it has been demonstrated that, for less trained personnel than the French and German at domesticating clean stems of oak, it might be hard to monitor epicormic shoot growth, which is under strong genetic control and hence susceptible to selective breeding [147]. Due to limited resources among forestry organizations and practitioners, successful tree breeding depends upon straightforward, reliable, and affordable methods of genetic improvement [147]. More efforts should be directed towards developing countries by finding international access to funds, research innovations, and training personnel to be able to manage and control crucial genetic improvement indicators.

#### 5.4.3. Cost Effectiveness

Growing genetically modified trees reduces the costs associated with chemicals for lignin removal for paper-making industries [23,57,148] also, a large quantity is saved as only a few amounts are enough for meeting the required demand for processing. Moreover, genetically modified poplar for insect resistance costs less when planting, as no more pesticides are required [12] and forest productivity will increase as there is no damage to the growth of the trees [148]. Despite that there is genetic improvement on the insect-resistant trees through biotechnology, the alteration might result in secondary pests, which might increase the management costs [148]. Through phytoremediation of trees, the lands that have been largely damaged and not used can be restored at lower costs while conserving the environment. For example, industries that dispose of harmful chemicals can make use of this method to use modified trees for absorbing these toxic chemicals instead of using expensive and destructive means [27,101,149].

#### 5.4.4. Inequality in the Forestry Sector

Genetically modified trees create gaps in the market as the wood from the improved variety costs more, leaving behind small mills [28]. For example, low-lignin wood will have more demand on the market for the pulp and paper industry, unlike the unimproved one. However, the price tag for the improved ones might be higher compared to that one form an unimproved tree. Innovations are highly owned by big companies, which are concentrated in China, the U.S, and Brazil [28]. Many countries lack the necessities for the development and deployment of improved tree varieties, thus creating continuous dependency on foreign technologies. High costs linked with intellectual property rights might exclude small stakeholders, companies, and institutions from utilizing genetically modified technology and trees [150]. Genetically improved trees might as well outcompete traditional varieties, resulting in dominating conventional trees [151]. Despite the economic value of genetically modified trees in the industry, wild trees still serve as the base for all the technologies and advancements achieved, and are highly protected, especially for the endangered species [139].

#### 5.4.5. Land Use

Genetically modified trees serve the land use efficiency as a massive productivity can be achieved in a limited area of land [148]. However, increasing demands for wood products might lead to the clearing of community lands and native species areas for the aim of expanding plantations of genetically improved varieties, which will lead to conflicts with society, but also will lead to loss of genetic biodiversity as more uniform plantations will dominate [148]. Due to these reasons, attention should be given to the allocation of plantations to avoid the gene escape from genetically modified tree plantations to the wild, as the effects are yet diverse. Also, there should be buffer zones to prevent contamination of wild types with the engineered trees.

## 6. Conclusions and Future Perspective

The genetic modification of trees has evolved from fundamental research to practical applications, addressing key challenges in forestry. Current advancements in growth enhancement, wood quality improvement, and stress tolerance highlight the potential of this technology. Researchers must apply innovations in genome editing, multi-omics analysis, and computational modeling to further accelerate progress in producing multi-trait tree varieties to fight against climate change. For example, fast-growing species can be further modified to provide multipurpose benefits such as biomass accumulation, phytoremediation, and resistance against both biotic and abiotic stresses. China’s experience demonstrates that consistent investment in tree genetic improvement leads to significant benefits such as enhanced wood quality, improved disease resistance, and increased growth rates [26]. With careful development and responsible implementation, GMTs could contribute to sustainable forest management, climate change mitigation through increased carbon sequestration, and help meet the rising global demand for timber, pulp, and bioenergy. Furthermore, GMTs offer the potential to restore degraded landscapes and improve biodiversity when integrated with ecological planning. In the coming decades, their applications are expected to extend beyond traditional forestry, reaching urban environments for space-efficient cultivation, and ecological restoration projects aimed at rehabilitating ecosystems affected by human activity and climate stress [152]. However, there are growing concerns about its potential impact on both the environment and human health, due to risks like unintended gene transfer, genetic instability, harm to non-target species, development of resistant pests and weeds, as well as possible toxicity and allergic reactions [29]. Since all the living organisms interact with each other in an ecosystem, the worry has been on the dispersal of the pollen seeds from the genetically modified trees to the wild and managed ecosystems could cause risks and unintended effects [121,153]. Developing guidelines for using GMTs in forestry should prioritize environmental protection and public concerns. Socio-economic studies and public acceptance of GMO policies are key. Strong collaboration between research and industry is essential to assess GMTs’ social and environmental impacts [121]. However, to fully harness the benefits of GMTs, it is essential to overcome technical challenges, establish effective regulatory frameworks, and enhance public awareness. Establishing effective regulatory frameworks is critical to ensure biosafety, traceability, and ethical oversight, especially when navigating international trade and cross-border environmental policies. Equally important is enhancing public awareness and engagement to build trust, address concerns about genetic modification, and promote informed dialog among stakeholders, including scientists, policymakers, industry leaders, and communities. Future research should prioritize developing climate-resilient tree varieties capable of withstanding extreme weather events, improving ecosystem services such as carbon sequestration, soil stabilization, and water regulation, and promoting biodiversity. Integrating emerging technologies like synthetic biology for precise genome editing and AI-assisted breeding for predictive trait selection will accelerate innovation and scalability. Multidisciplinary collaborations and long-term field trials will be essential to validate safety and performance while aligning GMTs’ development with global sustainability goals [154]. To ensure the long-term resilience of forest ecosystems, the future of forest tree breeding must embrace a comprehensive and integrated approach. This entails merging advanced genetic technologies such as genome editing, marker-assisted selection, and transgenic methods with a profound understanding of forest ecology and socio-economic dynamics. These elements must work in tandem to develop tree species that are not only high-performing but also resilient to the mounting pressures of global environmental change, including shifting climate patterns, emerging pests and diseases, and land use transformations. Moreover, successful tree breeding programs must consider the cultural and economic dimensions of forestry, aligning innovation with local needs and values. Incorporating community participation, indigenous knowledge, and public engagement into breeding initiatives will foster greater acceptance and equitable benefits. At the same time, the development of decision-support tools, sustainability metrics, and policy frameworks can help guide the responsible deployment of genetically enhanced trees. Investments in cross-disciplinary research, international collaboration, and transparent data sharing are key to addressing existing gaps. By adopting this holistic vision, the next generation of forest trees can offer improved productivity, ecological stability, and adaptability, ensuring forests remain vital contributors to biodiversity, climate regulation, and human well-being for generations to come.

## Figures and Tables

**Table 1:** Traditional breeding techniques.

Selective Breeding	Hybridization	Provenance Trial
Choosing superior trees from their natural stand based on their physical characteristics and collecting seeds for further breeding.	Trees from different genetic make-up or ancestry are crossed to obtain a recombinant variant, for example, a wood-improved species is crossed with a drought-tolerant type.	Seeds from different geographical locations are collected and planted under the same conditions, and the best-performing genotype in the environment will be further used for breeding purposes.

**Table 2:** Application of CRISPR in tree genome editing.

Species	Gene Edited	Trait Modified	Function	Reference
Poplar species	*CSE*	Wood quality improvement For biofuel production	Reduces lignin biosynthesis and enhances biomass quality	[13]
Poplar species	*PDS*	Participates in carotenoid biosynthesis and is used as a visual marker	Albino phenotype used to validate CRISPR editing	[50]
Poplar hybrid	*PagHyPRP1*	Drought and salt stress tolerance	Enable poplars to withstand water scarcity and a saline environment	[51]
*Populus tomesa*	*PtoMYB142*	Drought stress tolerance	Knockout improves drought tolerance of poplars	[52]
*Populus tomesa*	*PtoMYB156*	Positively regulates secondary cell wall formation	Knockout of *PtoMYB156* causes accumulation of lignin, cellulose, and Xylan	[53]
*Populus trichocarpa*	*PtHY5a*	Modulates seasonal growth	Cease growth during winter and promote bud development after winter break	[49]
*Populus trichocarpa*	*PtWRKY18* and *PtWRKY35*	Biotic stress resistance	Melampsora resistance	[54]
*Populus alba* × *P. glandulosa*	*PagGLR2.8*	Improves poplar wood quality	Knockout of *PagGLR2.8* enhances wood fiber quality	[55]
*Cryptomeria japonica* (L. f.) D. Don	*CjACOS5a* and *CjACOS5b*	Production of male sterile strobili	Knockout of *CjACOS5* causes the production of pollen-free male strobili	[56]

**Table 3:** Global status of genetically engineered tree field trials.

Country	Species	Applicant for Field Testing	Reference
China	Insect-resistant poplar species.	China	[12]
	Locust tree for insect resistance, drought tolerance, and improved wood quality.		www.fao.org
	Japanese pagoda for enhancing engineered for growth acceleration and stress tolerance.		[28]
	Bamboo species engineered for growth and development, and stress tolerance.		[123]
	Rubber tree (*Hevea brasiliensis* (Willd. ex A. Juss.) Müll. Arg.) engineered for high yield potential, disease resistance, and drought tolerance.		[28]
Malaysia	Genetically engineered rubber tree for pharmaceuticals.	Malaysian Rubber board	[124]
Sweden	Genetically engineered birch for growth performance and adaptability.	Universities	[125]
India	Genetically engineered rubber tree (*H. brasiliensis*) for fast growth.	Rubber research institute of India	[28]
United states of America	Loblolly *Pinus* spp. engineered for bioenergy, timber and pulp, and paper industries.	ArborGen	[126]
	Genetically engineered *Eucalyptus* spp. for cold tolerance.	Suzano, FuturaGene	[28]
	Insect-resistant American chestnut. fast growth poplar species engineered for carbon sequestration.	State University of New York, college of environmental science	
New Zealand	Genetically engineered radiata pine for herbicide tolerance, climate resilience, and drought tolerance.	SCION forest research institute. SCION forest research institute	[126]
	Genetically engineered Norway spruce for herbicide tolerance.		
Japan	Genetically engineered eucalyptus for cold tolerance.	Horizon 2 with ArborGen	[28]
	Genetically engineered eucalyptus for cold tolerance and salt tolerance.	University of Tsukuba’s gene research Centre	
	Genetically engineered Poplar for cellulose increment.	Forest tree breeding Centre	
	Pollen-free genetically engineered *Cryptomeria japonica*.		
	Genetically engineered eucalyptus tree to grow in acidic soils.	Japan’s Oji paper Company and Gifu University	[127]
Canada	Genetically engineered Poplar for herbicide tolerance and early flowering.	Canadian forest service	[28]

## Data Availability

No new data were created or analyzed in this study. Data sharing is not applicable to this article.

## References

[1] Harris N.L., Gibbs D.A., Baccini A., Birdsey R.A., de Bruin S., Farina M., Fatoyinbo L., Hansen M.C., Herold M., Houghton R.A. (2021). Global maps of twenty-first century forest carbon fluxes. Nat. Clim. Change.

[2] FAO (2020). Global Forest Resources Assessment 2020: Main Report.

[3] Upadhyay P., Singh T.S., Singh H. (2024). The Importance of Conserving Existing Forest Areas and Protecting Biodiversity in Addressing Climate Change Issues. Forests and Climate Change: Biological Perspectives on Impact, Adaptation, and Mitigation Strategies.

[4] Aerts R., Honnay O. (2011). Forest restoration, biodiversity and ecosystem functioning. BMC Ecol..

[5] Artaxo P., Hansson H.C., Machado L.A.T., Rizzo L.V. (2022). Tropical forests are crucial in regulating the climate on Earth. PLoS Clim..

[6] Harfouche A., Meilan R., Altman A. (2011). Tree genetic engineering and applications to sustainable forestry and biomass production. Trends Biotechnol..

[7] Singh N.R., Kamini, Kumar N., Kumar D., Jhariya M.K., Banerjee A., Meena R.S., Yadav D.K. (2019). Short-Rotation Forestry: Implications for Carbon Sequestration in Mitigating Climate Change. Sustainable Agriculture, Forest and Environmental Management.

[8] Manohar K.A., Shukla G., Shahina N.N., Sivasankarreddy K., Ravuther S.S., Chakravarty S., Thomas T.D., Razdan M.K., Kumar A. (2024). Conventional Versus Non-Conventional Methods of Propagation of Forest Tree Species: Applications and Limitations. Biotechnological Approaches for Sustaining Forest Trees and Their Products.

[9] Zhao Y., Tian Y., Sun Y., Li Y. (2022). The Development of Forest Genetic Breeding and the Application of Genome Selection and CRISPR/Cas9 in Forest Breeding. Forests.

[10] Ray D., Berlin M., Alia R., Sanchez L., Hynynen J., González-Martinez S., Bastien C. (2022). Transformative changes in tree breeding for resilient forest restoration. Front. For. Glob. Change.

[11] Avisara D., Dias T.B., dos Santos A.A., Galan M.P., Gonsalves J.M.W., Graca R.N., Livne S., Manoeli A., Drezza T.R., Porto A.C.M. (2023). Safety of genetically modified glyphosate-tolerant eucalyptus designed for integrated weed management. Adv. Weed Sci..

[12] Wang G.Y., Dong Y., Liu X.J., Yao G.S., Yu X.Y., Yang M.S. (2018). The Current Status and Development of Insect-Resistant Genetically Engineered Poplar in China. Front. Plant Sci..

[13] Jang H.A., Bae E.K., Kim M.H., Park S.J., Choi N.Y., Pyo S.W., Lee C., Jeong H.Y., Lee H., Choi Y.I. (2021). CRISPR-Knockout of *CSE* Gene Improves Saccharification Efficiency by Reducing Lignin Content in Hybrid Poplar. Int. J. Mol. Sci..

[14] Li Y., Yuan Y.H., Hu Z.J., Liu S.Y., Zhang X. (2024). Genetic Transformation of Forest Trees and Its Research Advances in Stress Tolerance. Forests.

[15] Ahmar S., Gill R.A., Jung K.-H., Faheem A., Qasim M.U., Mubeen M., Zhou W. (2020). Conventional and Molecular Techniques from Simple Breeding to Speed Breeding in Crop Plants: Recent Advances and Future Outlook. Int. J. Mol. Sci..

[16] Koskela J., Vinceti B., Dvorak W., Bush D., Dawson I.K., Loo J., Kjaer E.D., Navarro C., Padolina C., Bordacs S. (2014). Utilization and transfer of forest genetic resources: A global review. For. Ecol. Manag..

[17] Yang J., Liu Y., Liang B., Yang Q., Li X., Chen J., Li H., Lyu Y., Lin T. (2023). Genomic basis of selective breeding from the closest wild relative of large-fruited tomato. Hortic. Res..

[18] Benetka V., Novotná K., Štochlová P. (2014). Biomass production of *Populus nigra* L. clones grown in short station coppice systems in free different environments over four rotations. iForest-Biogeosci. For..

[19] Vujnović Z., Bogdan S., Lanscak M., Gavranović Markić A., Zorić N., Bogunović S., Ivankovic M. (2023). Preliminary Work on Generative Seedling Production and Clone Selection of European Black Poplar (*Populus nigra* L.). South-East Eur. For..

[20] Wang P.L., Si H., Li C.H., Xu Z.P., Guo H.M., Jin S.X., Cheng H.M. (2025). Plant genetic transformation: Achievements, current status and future prospects. Plant Biotechnol. J..

[21] Castellanos-Hernandez O.A., Acevedo-Hernández G., Rodriguez-Sahagun A., Herrera-Estrella L.R., Alvarez M.A. (2011). Genetic Transformation of Forest Trees. Genetic Transformation.

[22] Bhuvaneswari R., Saravanan K.R., Vennila S., Suganthi S. (2020). Precision Breeding Techniques: CRISPR-Cas9 and Beyond in Modern Plant Improvement. Plant Sci. Arch..

[23] Chanoca A., de Vries L., Boerjan W. (2019). Lignin Engineering in Forest Trees. Front. Plant Sci..

[24] Hasan N., Choudhary S., Naaz N., Sharma N., Laskar R.A. (2021). Recent advancements in molecular marker-assisted selection and applications in plant breeding programmes. J. Genet. Eng. Biotechnol..

[25] Liu D., Lei X.D., Gao W.Q., Guo H., Xie Y.S., Fu L.Y., Lei Y.C., Li Y.T., Zhang Z.L., Tang S.Z. (2022). Mapping the potential distribution suitability of 16 tree species under climate change in northeastern China using Maxent modelling. J. For. Res..

[26] Li X., Liu X.T., Wei J.T., Li Y., Tigabu M., Zhao X.Y. (2020). Genetic Improvement of *Pinus koraiensis* in China: Current Situation and Future Prospects. Forests.

[27] Kumar K., Shinde A., Aeron V., Verma A., Arif N.S. (2023). Genetic engineering of plants for phytoremediation: Advances and challenges. J. Plant Biochem. Biotechnol..

[28] Sharratt L., Chelo J. (2022). The Global Status of Genetically Engineered Tree Development: A Growing Threat.

[29] Kumar K., Gambhir G., Dass A., Tripathi A.K., Singh A., Jha A.K., Yadava P., Choudhary M., Rakshit S. (2020). Genetically modified crops: Current status and future prospects. Planta.

[30] Ozyigit I.I. (2020). Gene transfer to plants by electroporation: Methods and applications. Mol. Biol. Rep..

[31] Matthysse A.G., Dworkin M., Falkow S., Rosenberg E., Schleifer K.-H., Stackebrandt E. (2006). The Genus Agrobacterium. The Prokaryotes: Volume 5: Proteobacteria: Alpha and Beta Subclasses.

[32] Zhao H., Fu Y., Lv W., Zhang X., Li J., Yang D., Shi L., Wang H., Li W., Huang H. (2025). PuUBL5-mediated ZINC FINGER PROTEIN 1 stability is critical for root development under drought stress in *Populus ussuriensis*. Plant Physiol..

[33] Zhang J., Tang X., Sun M., Liu C., Yu H. (2025). A Genotype-Independent Transformation and Gene-Editing System for *Populus*. Plant Biotechnol. J..

[34] Hira M., Rubab Zahra N., Ammara M., Muhammad Waseem S., Raza S. (2016). Gene transformation: Methods, Uses and Applications. J. Pharm. Biol. Sci..

[35] Song C.W., Lu L., Guo Y.Y., Xu H.M., Li R.L. (2019). Efficient Agrobacterium-Mediated Transformation of the Commercial Hybrid Poplar *Populus Alba* × *Populus glandulosa* Uyeki. Int. J. Mol. Sci..

[36] Su W.B., Xu M.Y., Radani Y., Yang L.M. (2023). Technological Development and Application of Plant Genetic Transformation. Int. J. Mol. Sci..

[37] Arokiaraj P., Jones H., Cheong K.F., Coomber S., Charlwood B.V. (1994). Gene insertion into *Hevea brasiliensis*. Plant Cell Rep..

[38] Noël A., Levasseur C., Le V.Q., Séguin A. (2005). Enhanced resistance to fungal pathogens in forest trees by genetic transformation of black spruce and hybrid poplar with a *Trichoderma harzianum* endochitinase gene. Physiol. Mol. Plant Pathol..

[39] Mohan S., Khan A. (2024). Marker Assisted Selection: Concept and Role in Crop Improvement. Advances in Genetics and Plant Breeding.

[40] Remington D.L., Whetten R.W., Liu B.H., O’Malley D.M. (1999). Construction of an AFLP genetic map with nearly complete genome coverage in *Pinus taeda*. Theor. Appl. Genet..

[41] Chandra K., Pandey S., Chand S., Kumawat G., Kumawat C.K., Mishra U.N., Sharma R., Lenka D., Abdurakhmonov I.Y. (2020). Insights into Marker Assisted Selection and Its Applications in Plant Breeding. Plant Breeding—Current and Future Views.

[42] Parida A.K., Jha B. (2010). Salt tolerance mechanisms in mangroves: A review. Trees.

[43] Zhou X., Zhang L., Zhang M., Wei H., Bai Y., Tian J., Hu J. (2025). Genomic selection for growth and wood properties in multi-generation hybrid populations of *Populus deltoides*. Hortic. Res..

[44] Tsarouhas V., Gullberg U., Lagercrantz U. (2004). Mapping of Quantitative Trait Loci (qtls) Affecting Autumn Freezing Resistance and Phenology in *Salix*. Theor. Appl. Genet..

[45] Karlson C.K.S., Mohd-Noor S.N., Nolte N., Tan B.C. (2021). CRISPR/dCas9-Based Systems: Mechanisms and Applications in Plant Sciences. Plants.

[46] Doudna J.A., Charpentier E. (2014). The new frontier of genome engineering with CRISPR-Cas9. Science.

[47] Cabelkova I., Sanova P., Hlavacek M., Broz D., Smutka L., Prochazka P. (2024). The moderating role of perceived health risks on the acceptance of genetically modified food. Front. Public Health.

[48] Tang H., Yuan H., Du W., Li G., Xue D., Huang Q. (2021). Active-Site Models of *Streptococcus pyogenes* Cas9 in DNA Cleavage State. Front. Mol. Biosci..

[49] Gao Y., Chen Z., Feng Q., Long T., Ding J., Shu P., Deng H., Yu P., Tan W., Liu S. (2024). ELONGATED HYPOCOTYL 5a modulates *FLOWERING LOCUS T2* and gibberellin levels to control dormancy and bud break in poplar. Plant Cell.

[50] Bae E.-K., Choi H., Choi J.W., Lee H., Kim S.-G., Ko J.-H., Choi Y.-I. (2021). Efficient knockout of the phytoene desaturase gene in a hybrid poplar (*Populus alba* × *Populus glandulosa*) using the CRISPR/Cas9 system with a single gRNA. Transgenic Res..

[51] Zhang T., Zhang W., Ding C., Hu Z., Fan C., Zhang J., Li Z., Diao S., Shen L., Zhang B. (2023). A breeding strategy for improving drought and salt tolerance of poplar based on CRISPR/Cas9. Plant Biotechnol. J..

[52] Song Q., Kong L., Yang J., Lin M., Zhang Y., Yang X., Wang X., Zhao Z., Zhang M., Pan J. (2024). The transcription factor PtoMYB142 enhances drought tolerance in *Populus tomentosa* by regulating gibberellin catabolism. Plant J..

[53] Yang L., Zhao X., Ran L., Li C., Fan D., Luo K. (2017). PtoMYB156 is involved in negative regulation of phenylpropanoid metabolism and secondary cell wall biosynthesis during wood formation in poplar. Sci. Rep..

[54] Jiang Y., Guo L., Ma X., Zhao X., Jiao B., Li C., Luo K. (2017). The WRKY transcription factors *PtrWRKY18* and *PtrWRKY35* promote *Melampsora* resistance in *Populus*. Tree Physiol..

[55] An Y., Wang S.-Q., Jia X.-Y., Jiao X., Qu M.-Q., Dong Y., Wang Z.-Y., Ma Z.-Y., Yang S., Han X. (2025). Bioengineered poplar fibres via *PagGLR2.8* editing: A synergistic design for high-performance biocomposites. Plant Biotechnol. J..

[56] Nishiguchi M., Futamura N., Endo M., Mikami M., Toki S., Katahata S.-I., Ohmiya Y., Konagaya K.-I., Nanasato Y., Taniguchi T. (2023). CRISPR/Cas9-mediated disruption of *CjACOS5* confers no-pollen formation on sugi trees (*Cryptomeria japonica* D. Don). Sci. Rep..

[57] Ralph J., Lapierre C., Boerjan W. (2019). Lignin structure and its engineering. Curr. Opin. Biotechnol..

[58] Mandal D.D., Singh G., Majumdar S., Chanda P. (2023). Challenges in developing strategies for the valorization of lignin-a major pollutant of the paper mill industry. Environ. Sci. Pollut. Res..

[59] Yang X., Lin Q., Udayabhanu J., Hua Y., Dai X., Xin S., Wang X., Huang H., Huang T. (2024). An optimized CRISPRCas9-based gene editing system for efficiently generating homozygous edited plants in rubber tree (*Hevea brasiliensis*). Ind. Crops Prod..

[60] Ricroch A., Eriksson D., Miladinović D., Sweet J., Van Laere K., Woźniak-Gientka E. (2024). A Roadmap for Plant Genome Editing.

[61] Son S., Park S.R. (2022). Challenges Facing CRISPR/Cas9-Based Genome Editing in Plants. Front. Plant Sci..

[62] Plomion C., Bastien C., Bogeat-Triboulot M.-B., Bouffier L., Déjardin A., Duplessis S., Fady B., Heuertz M., Le Gac A.-L., Le Provost G. (2016). Forest tree genomics: 10 achievements from the past 10 years and future prospects. Ann. For. Sci..

[63] Cárdenas-Zapata R., Palma-Ramírez D., Flores-Vela A.I., Romero-Partida J.N., Paredes-Rojas J.C., Márquez-Rocha F.J., Bravo-Díaz B. (2022). Structural and thermal study of hemicellulose and lignin removal from two types of sawdust to isolate cellulose. MRS Adv..

[64] Kienberger M., Krozer Y., Narodoslawsky M. (2019). Potential Applications of Lignin. Economics of Bioresources: Concepts, Tools, Experiences.

[65] Nargotra P., Sharma V., Wang H.M.D., Shieh C.J., Liu Y.C., Kuo C.H. (2025). Biocatalysis for Lignin Conversion and Valorization: Driving Sustainability in the Circular Economy. Catalysts.

[66] Yan J., Aznar A., Chalvin C., Birdseye D.S., Baidoo E.E.K., Eudes A., Shih P.M., Loqué D., Zhang A., Scheller H.V. (2018). Increased drought tolerance in plants engineered for low lignin and low xylan content. Biotechnol. Biofuels.

[67] Wilkerson C.G., Mansfield S.D., Lu F., Withers S., Park J.Y., Karlen S.D., Gonzales-Vigil E., Padmakshan D., Unda F., Rencoret J. (2014). Monolignol Ferulate Transferase Introduces Chemically Labile Linkages into the Lignin Backbone. Science.

[68] Sulis D.B., Jiang X., Yang C., Marques B.M., Matthews M.L., Miller Z., Lan K., Cofre-Vega C., Liu B., Sun R. (2023). Multiplex CRISPR editing of wood for sustainable fiber production. Science.

[69] Ha C.M., Rao X., Saxena G., Dixon R.A. (2021). Growth-defense trade-offs and yield loss in plants with engineered cell walls. New Phytol..

[70] Beckers B., Op De Beeck M., Weyens N., Van Acker R., Van Montagu M., Boerjan W., Vangronsveld J. (2016). Lignin engineering in field-grown poplar trees affects the endosphere bacterial microbiome. Proc. Natl. Acad. Sci. USA.

[71] Farooq M., Hussain M., Wahid A., Siddique K.H.M., Aroca R. (2012). Drought Stress in Plants: An Overview. Plant Responses to Drought Stress: From Morphological to Molecular Features.

[72] Polle A., Chen S.L., Eckert C., Harfouche A. (2019). Engineering Drought Resistance in Forest Trees. Front. Plant Sci..

[73] Lawlor D.W. (2013). Genetic engineering to improve plant performance under drought: Physiological evaluation of achievements, limitations, and possibilities. J. Exp. Bot..

[74] Pamungkas S.T.P., Suwarto, Suprayogi, Farid N. (2022). Drought Stress: Responses and Mechanism in Plants. Rev. Agric. Sci..

[75] Wang C., Zhou X., Qin J., Wang X., Yu H., Zhu Q., Lee D., Chen L. (2024). Comprehensive Response Mechanisms of Plants to Water Deficit: A Physiological, Biochemical, Molecular, and Ecological Review. Mol. Soil. Biol..

[76] Zang R., Zhang Q., Shao M.a., Jia X., Wei X. (2017). Relationship of Climatic and Forest Factors to Drought- and Heat-Induced Tree Mortality. PLoS ONE.

[77] Fidler J., Graska J., Gietler M., Nykiel M., Prabucka B., Rybarczyk-Plonska A., Muszynska E., Morkunas I., Labudda M. (2022). PYR/PYL/RCAR Receptors Play a Vital Role in the Abscisic-Acid-Dependent Responses of Plants to External or Internal Stimuli. Cells.

[78] Schweighofer A., Hirt H., Meskiene L. (2004). Plant PP2C phosphatases: Emerging functions in stress signaling. Trends Plant Sci..

[79] Son S., Park S.R. (2023). The rice SnRK family: Biological roles and cell signaling modules. Front. Plant Sci..

[80] Singh D., Laxmi A. (2015). Transcriptional regulation of drought response: A tortuous network of transcriptional factors. Front. Plant Sci..

[81] Shinwari Z.K., Jan S.A., Nakashima K., Yamaguchi-Shinozaki K. (2020). Genetic engineering approaches to understanding drought tolerance in plants. Plant Biotechnol. Rep..

[82] Martignago D., Rico-Medina A., Blasco-Escáez D., Fontanet-Manzaneque J.B., Cano-Delgado A.I. (2020). Drought Resistance by Engineering Plant Tissue-Specific Responses. Front. Plant Sci..

[83] Li Z.X., Zhang J.R., Song X.Y. (2024). Breeding Maize Hybrids with Improved Drought Tolerance Using Genetic Transformation. Int. J. Mol. Sci..

[84] Zahedi S.M., Karimi M., Venditti A., Zahra N., Siddique K.H.M., Farooq M. (2025). Plant Adaptation to Drought Stress: The Role of Anatomical and Morphological Characteristics in Maintaining the Water Status. J. Soil Sci. Plant Nutr..

[85] Yang X., Lu M., Wang Y., Wang Y., Liu Z., Chen S. (2021). Response Mechanism of Plants to Drought Stress. Horticulturae.

[86] Kou X.Y., Han W.H., Kang J. (2022). Responses of root system architecture to water stress at multiple levels: A meta-analysis of trials under controlled conditions. Front. Plant Sci..

[87] Brandt B., Munemasa S., Wang C., Desiree N., Yong T., Yang P.G., Poretsky E., Belknap T.F., Waadt R., Aleman F. (2015). Calcium specificity signaling mechanisms in abscisic acid signal transduction in *Arabidopsi* guard cells. eLife.

[88] Aliniaeifard S., Shomali A., Seifikalhor M., Lastochkina O., Hasanuzzaman M., Tanveer M. (2020). Calcium Signaling in Plants Under Drought. Salt and Drought Stress Tolerance in Plants: Signaling Networks and Adaptive Mechanisms.

[89] Mu Z.Y., Xu M.Y., Manda T., Yang L.M., Hwarari D., Zhu F.Y. (2024). Genomic survey and evolution analysis of calcium-dependent protein kinases in plants and their stress-responsive patterns in populus. BMC Genom..

[90] Anderson J.P., Badruzsaufari E., Schenk P.M., Manners J.M., Desmond O.J., Ehlert C., Maclean D.J., Ebert P.R., Kazan K. (2004). Antagonistic interaction between abscisic acid and jasmonate-ethylene signaling pathways modulates defense gene expression and disease resistance in *Arabidopsis*. Plant Cell.

[91] Kaya C., Ugurlar F., Adamakis I.D.S. (2024). Epigenetic Modifications of Hormonal Signaling Pathways in Plant Drought Response and Tolerance for Sustainable Food Security. Int. J. Mol. Sci..

[92] Liu S.J., Zhang H., Jin X.T., Niu M.X., Feng C.H., Liu X., Chao L., Wang H.-L., Yin W., Xia X. (2024). PeFUS3 Drives Lateral Root Growth Via Auxin and ABA Signalling Under Drought Stress in *Populus*. Plant Cell Environ..

[93] Shen C., Zhang Y., Li Q., Liu S., He F., An Y., Zhou Y., Liu C., Yin W., Xia X. (2021). PdGNC confers drought tolerance by mediating stomatal closure resulting from NO and H_2_O_2_ production via the direct regulation of *PdHXK1* expression in Populus. New Phytol..

[94] Uma G.S., Mahanta D.K., Sharma L., Thomas T.D., Razdan M.K., Kumar A. (2024). Advancing Forest Insect Pest Management: A Focus on Biotechnological Approaches. Biotechnological Approaches for Sustaining Forest Trees and Their Products.

[95] Gao B., Zhu S. (2024). The evolutionary novelty of insect defensins: From bacterial killing to toxin neutralization. Cell. Mol. Life Sci..

[96] Wei T. (2003). Recent advances in the molecular genetics of resin biosynthesis and genetic engineering strategies to improve defenses in conifers. J. For. Res..

[97] Woodcock P., Cottrell J.E., Buggs R.J.A., Quine C.P. (2018). Mitigating pest and pathogen impacts using resistant trees: A framework and overview to inform development and deployment in Europe and North America. Forestry.

[98] Pinheiro A.C., Dos Santos A.A., Avisar D., Gonsalves J.M., Galan M.P., Abramson M., Barimboim N., Abrahao O., Graca R.N., Drezza T.R. (2023). Five-years Post Commercial Approval Monitoring of Eucalyptus H421. Front. Bioeng. Biotechnol..

[99] Sharma M., Rawat S., Rautela A. (2024). Phytoremediation in sustainable wastewater management: An eco-friendly review of current techniques and future prospects. Aqua-Water Infrastruct. Ecosyst. Soc..

[100] Ahmad H., Abd El-Rahim W.M. (2024). Phytoremediation: The Green Solution. Bioremediation for Environmental Sustainability.

[101] Ozyigit I.I., Can H., Dogan I. (2021). Phytoremediation using genetically engineered plants to remove metals: A review. Environ. Chem. Lett..

[102] Bamagoos A.A., Mallhi Z.I., El-Esawi M.A., Rizwan M., Ahmad A., Hussain A., Alharby H.F., Alharbi B.M., Ali S. (2022). Alleviating lead-induced phytotoxicity and enhancing the phytoremediation of castor bean (*Ricinus communi* L.) by glutathione application: New insights into the mechanisms regulating antioxidants, gas exchange and lead uptake. Int. J. Phytoremediation.

[103] Ionata E., Caputo E., Mandrich L., Marcolongo L. (2024). Moving towards Biofuels and High-Value Products through Phytoremediation and Biocatalytic Processes. Catalysts.

[104] Park J.K., Oh K. (2023). Advancements in Phytoremediation Research for Soil and Water Resources: Harnessing Plant Power for Environmental Cleanup. Sustainability.

[105] Koul B., Taak P., Koul B., Taak P. (2018). Chemical Methods of Soil Remediation. Biotechnological Strategies for Effective Remediation of Polluted Soils.

[106] Lone M.I., He Z.L., Stoffella P.J., Yang X.E. (2008). Phytoremediation of heavy metal polluted soils and water: Progresses and perspectives. J. Zhejiang Univ.-Sci. B.

[107] Mukherjee A., Agrawal S.B., Agrawal M., Singh A., Prasad S.M., Singh R.P. (2016). Heavy Metal Accumulation Potential and Tolerance in Tree and Grass Species. Plant Responses to Xenobiotics.

[108] Reeves R.D., Baker A.J.M., Jaffré T., Erskine P.D., Echevarria G., van der Ent A. (2018). A global database for plants that hyperaccumulate metal and metalloid trace elements. New Phytol..

[109] Asare M.O., Szakova J., Tlustos P. (2023). Mechanisms of As, Cd, Pb, and Zn hyperaccumulation by plants and their effects on soil microbiome in the rhizosphere. Front. Environ. Sci..

[110] Kumar S., Shah S.H., Vimala Y., Jatav H.S., Ahmad P., Chen Y., Siddique K.H.M. (2022). Abscisic acid: Metabolism, transport, crosstalk with other plant growth regulators, and its role in heavy metal stress mitigation. Front. Plant Sci..

[111] Mihucz V.G., Csog Á., Fodor F., Tatár E., Szoboszlai N., Silaghi-Dumitrescu L., Záray G. (2012). Impact of two iron(III) chelators on the iron, cadmium, lead and nickel accumulation in poplar grown under heavy metal stress in hydroponics. J. Plant Physiol..

[112] Laureysens I., Blust R., De Temmerman L., Lemmens C., Ceulemans R. (2004). Clonal variation in heavy metal accumulation and biomass production in a poplar coppice culture: I. Seasonal variation in leaf, wood and bark concentrations. Environ. Pollut..

[113] Elobeid M., Göbel C., Feussner I., Polle A. (2012). Cadmium interferes with auxin physiology and lignification in poplar. J. Exp. Bot..

[114] Kumar K., Das D., Bhanage B.M., Arai M. (2014). Carbon Dioxide Sequestration by Biological Processes. Transformation and Utilization of Carbon Dioxide.

[115] Kumar A., Singh R., Chaurasia S. (2024). Carbon Sequestration by Trees in University Campus. J. Emerg. Technol. Innov. Res..

[116] Özparpucu M., Rüggeberg M., Gierlinger N., Cesarino I., Vanholme R., Boerjan W., Burgert I. (2017). Unravelling the impact of lignin on cell wall mechanics: A comprehensive study on young poplar trees downregulated for CINNAMYL ALCOHOL DEHYDROGENASE (CAD). Plant J..

[117] Finér L., Helmisaari H.-S., Lõhmus K., Majdi H., Brunner I., Børja I., Eldhuset T., Godbold D., Grebenc T., Konôpka B. (2007). Variation in fine root biomass of three European tree species: Beech (*Fagus sylvatica* L.), Norway spruce (*Picea abies* L. Karst.), and Scots Pine (*Pinus sylvestris* L.). Plant Biosyst..

[118] da Silva P.H.M., Abrahão O.S. (2021). Gene flow and spontaneous seedling establishment around genetically modified eucalypt plantations. New For..

[119] Donahue R.A., Davis T.D., Michler C.H., Riemenschneider D.E., Carter D.R., Marquardt P.E., Sankhla N., Sankhla D., Haissig B.E., Isebrands J.G. (1994). Growth, photosynthesis, and herbicide tolerance of genetically modified hybrid poplar. Can. J. For. Res..

[120] Hong-yu R., Ying C., Min-ren H., Ming-xiu W., Ning-feng W., Yun-liu F. (2000). Genetic Transformation of Poplar NL-80106 Transferred by Bt Gene and Its Insect-Resistance. J. Plant Resour. Environ..

[121] Nonic M., Vettori C., Boscaleri F., Milovanovic J., Sijacic-Nikolic M. (2012). Genetically modified trees: State and perspectives. Genetika.

[122] Grattapaglia D., Silva-Junior O.B., Resende R.T., Cappa E.P., Muller B.S.F., Tan B., Isik F., Ratcliffe B., El-Kassaby Y.A. (2018). Quantitative Genetics and Genomics Converge to Accelerate Forest Tree Breeding. Front. Plant Sci..

[123] Wang W.J., Wu Q.Y., Wang N.N., Ye S.W., Wang Y.J., Zhang J., Lin C.T., Zhu Q. (2025). Advances in bamboo genomics: Growth and development, stress tolerance, and genetic engineering. J. Integr. Plant Biol..

[124] Elumalai S. (2010). Genetic Transformation of Rubber Trees for Production of Pharmaceuticals and Improvement of Agronomic Traits. Ph.D. Thesis.

[125] Liziniewicz M., Barbeito I., Zvirgzdins A., Stener L.G., Niemistö P., Fahlvik N., Johansson U., Karlsson B., Nilsson U. (2022). Production of genetically improved silver birch plantations in southern and central Sweden. Silva Fenn..

[126] Sabatia C.O. (2011). Stand Dynamics, Growth, and Yield of Genetically Enhanced Loblolly Pine (*Pinus taeda* L.). Ph.D. Thesis.

[127] Doi T., Suzuki Y., Kobayashi Y., Kawazu T., Koyama H., Sato S. (2009). Improvement of root function to enhance growth of Eucalyptus. Jpn. For. Soc. Congr..

[128] Haygood R., Ives A.R., Andow D.A. (2004). Population genetics of transgene containment. Ecol. Lett..

[129] Slavov G.T., Leonardi S., Burczyk J., Adams W.T., Strauss S.H., Difazio S.P. (2009). Extensive pollen flow in two ecologically contrasting populations of *Populus trichocarpa*. Mol. Ecol..

[130] Zhang C., Norris-Caneda K.H., Rottmann W.H., Gulledge J.E., Chang S., Kwan B.Y.-H., Thomas A.M., Mandel L.C., Kothera R.T., Victor A.D. (2012). Control of Pollen-Mediated Gene Flow in Transgenic Trees. Plant Physiol..

[131] Merkle S. (2006). Engineering Forest Trees with Heavy Metal Resistance Genes. Silvae Genet..

[132] James D., Thomas T.D., Razdan M.K., Kumar A. (2024). Impact Assessment of Genetically Engineered Trees: An Overview on Risk Assessment and Management. Biotechnological Approaches for Sustaining Forest Trees and Their Products.

[133] Wang S., Liu J., Dong Y., Li Y., Huang Y., Ren M., Yang M., Wang J. (2022). Dynamic monitoring of the impact of insect-resistant transgenic poplar field stands on arthropod communities. For. Ecol. Manag..

[134] Xu P., Kong Y.M., Song D.L., Huang C., Li X., Li L.G. (2014). Conservation and functional influence of alternative splicing in wood formation of *Populus* and *Eucalyptus*. BMC Genom..

[135] Fan J., Dong Y., Yu X., Yao L., Li D., Wang J., Yang M. (2020). Assessment of environmental microbial effects of insect-resistant transgenic *Populus* × *euramericana* cv. ‘74/76’ based on high-throughput sequencing. Acta Physiol. Plant..

[136] Zheng Y., Lv G.B., Chen K., Yu Q., Niu B., Jiang J., Liu G. (2022). Impact of *PaGLK* transgenic poplar on microbial community and soil enzyme activity in rhizosphere soil. Front. Bioeng. Biotechnol..

[137] Kuparinen A., Schurr F.M. (2008). Assessing the risk of gene flow from genetically modified trees carrying mitigation transgenes. Biol. Invasions.

[138] Fritsche S., Klocko A.L., Boron A., Brunner A.M., Thorlby G. (2018). Strategies for Engineering Reproductive Sterility in Plantation Forests. Front. Plant Sci..

[139] World Intellectual Property Organization (2024). Intellectual Property and New Genomic Technologies: Proposals to Facilitate Identification, Access, and Use of Intellectual Property.

[140] Myking T., Walløe Tvedt M., Karlsson B. (2017). Protection of forest genetic resources by intellectual property rights—Exploring possibilities and conceivable conflicts. Scand. J. For. Res..

[141] Wright B.D. (2006). Plant Genetic Engineering and Intellectual Property Protection.

[142] Kushwaha N. (2025). Application of Genetic Engineering Technology in Trees: Innovations, Challenges and Future Prospects. J. Biochem. Int..

[143] De Meester B., Vanholme R., Mota T., Boerjan W. (2022). Lignin engineering in forest trees: From gene discovery to field trials. Plant Commun..

[144] Klopfenstein N.B., Chun Y.W., Kim M.S., Ahuja M.A., Dillon M.C., Carman R.C., Eskew L.G. (1997). Micropropagation, Genetic Engineering, and Molecular Biology of Populus.

[145] Hagen K., Engelhard M., Otto M., Simon S., Stracke K. (2022). Genetic Engineering, Nature Conservation and Biological Diversity: Boundaries of Design.

[146] Abbas M.S.T. (2018). Genetically engineered (modified) crops (*Bacillus thuringiensis* crops) and the world controversy on their safety. Egypt. J. Biol. Pest Control.

[147] Savill P., Kanowski P. (1993). Tree improvement programs for European oaks: Goals and strategies. Ann. Sci. For..

[148] FAO The Potential Environmental, Cultural and Socio-Economic Impacts of Genetically Modified Trees. Proceedings of the 13th meeting of the Subsidiary Body on Scientific, Technical and Technological Advice (SBSTTA).

[149] Ahmad H. (2024). Phytoremediation: The Green Solution.

[150] Steinbrecher R.A., Lorch A. Federation of German Scientists Genetically Engineered Trees & Risk Assessment. Proceedings of the 9th Conference of the Parties (COP9).

[151] Petermann A. (2005). International Status of GE Trees: UN FAO Report The International Status of Genetically Modified Trees.

[152] Yin Y., Wang C., Xiao D., Liang Y., Wang Y. (2021). Advances and Perspectives of Transgenic Technology and Biotechnological Application in Forest Trees. Front. Plant Sci..

[153] Megalos M.A., Williams C.G. (2006). Private Forests and Transgenic Forest Trees. Landscapes, Genomics and Transgenic Conifers.

[154] Bruegmann T., Fendel A., Zahn V., Fladung M., Ricroch A., Eriksson D., Miladinović D., Sweet J., Van Laere K., Woźniak-Gientka E. (2024). Genome Editing in Forest Trees. A Roadmap for Plant Genome Editing.

